# Reward Association Enhances Stimulus-Specific Representations in Primary Visual Cortex

**DOI:** 10.1016/j.cub.2020.03.018

**Published:** 2020-05-18

**Authors:** Julia U. Henschke, Evelyn Dylda, Danai Katsanevaki, Nathalie Dupuy, Stephen P. Currie, Theoklitos Amvrosiadis, Janelle M.P. Pakan, Nathalie L. Rochefort

**Affiliations:** 1 Center for Behavioral Brain Sciences, Institute of Cognitive Neurology and Dementia Research, Otto-von-Guericke University Magdeburg, Leipziger Str. 44, Magdeburg 39120, Germany; 2 German Center for Neurodegenerative Diseases, Leipziger Str. 44, Magdeburg 39120, Germany; 3 Centre for Discovery Brain Sciences, Edinburgh Medical School: Biomedical Sciences, University of Edinburgh, 15 George Square, Edinburgh, EH8 9XD, UK; 4 Simons Initiative for the Developing Brain, University of Edinburgh, 15 George Square, Edinburgh EH8 9XD, UK

**Keywords:** visual cortex, reward, reinforcement learning, orientation selectivity, plasiticy, awake mouse, layer 2/3, locomotion, visuomotor, stimulus discrimination

## Abstract

The potential for neuronal representations of external stimuli to be modified by previous experience is critical for efficient sensory processing and improved behavioral outcomes. To investigate how repeated exposure to a visual stimulus affects its representation in mouse primary visual cortex (V1), we performed two-photon calcium imaging of layer 2/3 neurons and assessed responses before, during, and after the presentation of a repetitive stimulus over 5 consecutive days. We found a stimulus-specific enhancement of the neuronal representation of the repetitively presented stimulus when it was associated with a reward. This was observed both after mice actively learned a rewarded task and when the reward was randomly received. Stimulus-specific enhanced representation resulted both from neurons gaining selectivity and from increased response reliability in previously selective neurons. In the absence of reward, there was either no change in stimulus representation or a decreased representation when the stimulus was viewed at a fixed temporal frequency. Pairing a second stimulus with a reward led to a similar enhanced representation and increased discriminability between the equally rewarded stimuli. Single-neuron responses showed that separate subpopulations discriminated between the two rewarded stimuli depending on whether the stimuli were displayed in a virtual environment or viewed on a single screen. We suggest that reward-associated responses enable the generalization of enhanced stimulus representation across these V1 subpopulations. We propose that this dynamic regulation of visual processing based on the behavioral relevance of sensory input ultimately enhances and stabilizes the representation of task-relevant features while suppressing responses to non-relevant stimuli.

## Highlights

• Reward association drives the enhanced representation of repetitive visual stimuli
• Increased discriminability between two equally rewarded stimuli with learning
• Enhanced responses to rewarded stimuli generalize across subpopulations and contexts
• Dynamic regulation of V1 responses based on behavioral relevance of visual input

## Teaser

Henschke et al. show that reward association enhances the representation of repetitive visual stimuli in mouse primary visual cortex. Two equally rewarded stimuli led to similar enhanced representation and increased discriminability with learning. Responses to rewarded stimuli generalize across V1 subpopulations and viewing contexts.

## Introduction

Adaptation to the environment is vital for survival and relies on our ability to selectively integrate relevant sensory information and ignore irrelevant distractors. This ability depends on the potential for neuronal networks to change through experience, for example, by learning the association of a specific sensory stimulus with a reward. Neuronal representations of visual stimuli in adult primary visual cortex (V1) have been shown to be modified by previous visual experience [1, 2, 3, 4, 5, 6, 7, 8, 9, 10, 11, 12, 13, 14, 15, 16, 17]. However, previous studies in mice have reported inconsistent results regarding the effect of a repetitively viewed stimulus under various behavioral conditions. Several studies have reported a stimulus-specific response potentiation to the daily presentation of a given stimulus [7, 8, 9, 10] or stimulus sequence [6] without any associated reward or aversive stimuli. Conversely, other studies have shown a stimulus-specific adaptation resulting in response suppression for repetitive stimuli [5]. In addition, studies that involved active learning tasks, where a specific stimulus was repetitively paired with an associated behavioral outcome, have shown either an increase in stimulus discriminability of behaviorally relevant stimuli [3, 4, 12, 16] or a stimulus-specific decrease in the number of visually responsive neurons and decreased stimulus selectivity [5]. Therefore, the extent to which the presentation of a visual stimulus can, by itself, lead to plasticity in V1 and alter subsequent neuronal responses to that stimulus remains unclear.

Part of this inconsistency may stem from the behavioral relevance of the stimulus and/or the behavioral state of the animal when viewing a visual stimulus. A number of factors can contribute to the salience of a visual stimulus and therefore its behavioral relevance [13], for example, whether a given stimulus is associated with a reward [17, 18, 19] or an aversive event [5] and whether this association was learned during a behavioral task (e.g., goal-directed behavior) [3, 4, 20]. Additionally, the behavioral state of an animal, such as whether the animal is stationary or running, modulates the activity of individual neurons in V1 [21, 22, 23, 24, 25, 26, 27, 28, 29] and affects the representation of visual stimuli [30, 31] (but see [32]). While moving through an environment, congruence between an animal’s self-motion and optic-flow information results in coupled visuomotor feedback; recent research has revealed that a subpopulation of neurons in V1 responds to a mismatch between optic-flow and self-motion signals when this visuomotor feedback is uncoupled [33, 34, 35, 36]. In order to detect salient stimuli and ignore irrelevant input, one may predict that neural circuits in the visual cortex could selectively reduce responses to distractors, increase their responses to visual stimuli that are relevant for a behavioral task, and remain unaffected by the natural optic flow associated with self-motion. However, how information regarding the behavioral relevance of a specific stimulus is encoded in the visual cortex and modified by experience remains uncertain.

In this study, we performed two-photon calcium imaging of V1 layer 2/3 neurons in awake head-fixed mice to assess neuronal responses before, during, and after the presentation of a single repetitive visual stimulus for 5 consecutive days. We systematically assessed how stimulus-reward association and visuomotor coupling affected the representation of the repetitive stimulus. We found a stimulus-specific enhancement of the neuronal representation of the repetitive stimulus when it was associated with a reward. When a second stimulus was subsequently associated with the same reward, we found a similar stimulus-specific increase, indicating that layer 2/3 neuronal populations can simultaneously maintain multiple enhanced representations of rewarded stimuli. In the absence of reward, there was either no change or a decrease in repeated stimulus representation over days. Single-cell responses showed that, although one V1 subpopulation selectively discriminated between rewarded stimuli in the virtual environment, a largely non-overlapping subpopulation enhanced their selectivity and discriminability for the rewarded orientations during single-screen viewing. We propose permissive mechanisms for reward responses to drive generalization across V1 populations and enhance stimulus representation. Altogether, these results support the view of a dynamic regulation of visual information processing in V1 based on the behavioral relevance of the visual input.

## Results

**Figure 1:**
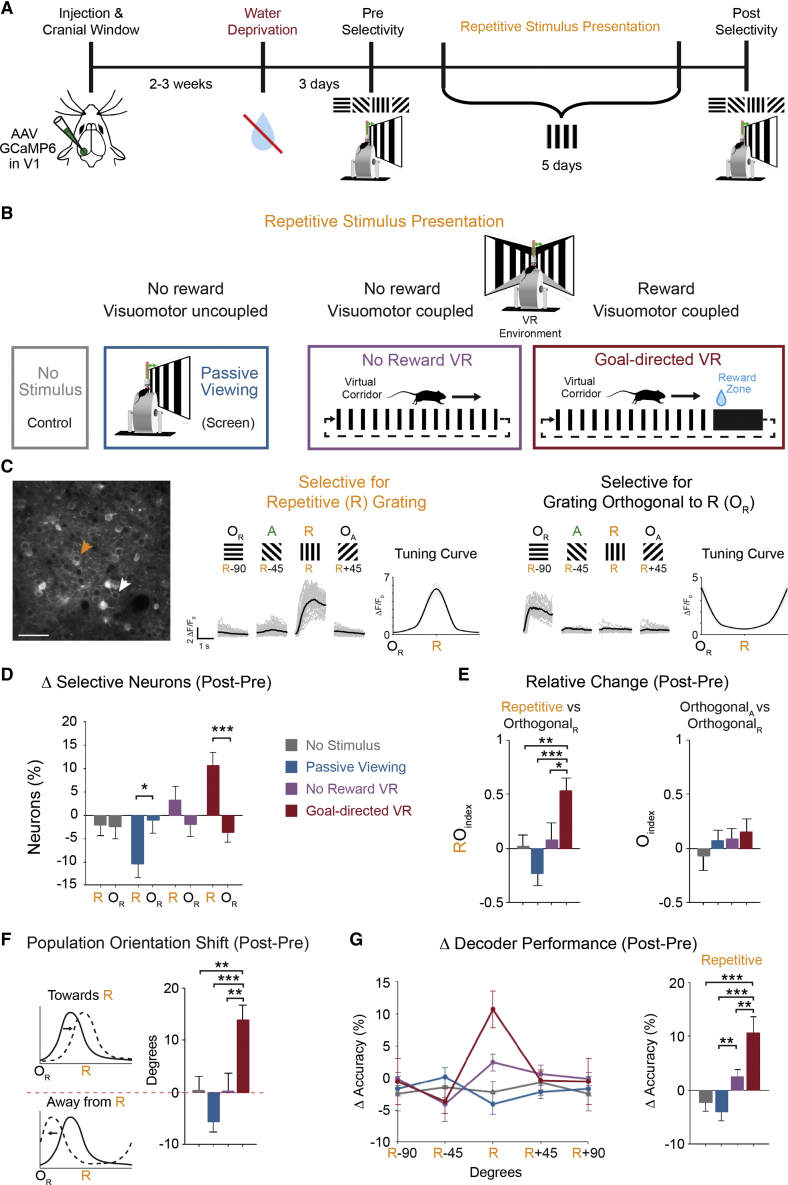
Enhanced or Attenuated Representation of a Repetitive Stimulus in V1 Layer 2/3 Neurons (A) Experimental timeline. Layer 2/3 neurons in V1 were imaged in head-fixed mice able to freely run on a cylindrical treadmill, and responses to 4 oriented gratings were assessed before (pre) and after (post) an intervening 5 consecutive days of repetitive stimulus presentation of one oriented grating. (B) During the repetitive visual stimulus over the 5 days, animals were divided into experimental groups: *no stimulus* presentation; *passive viewing* condition with one grating at a constant temporal frequency (schematic of setup with single screen); and same grating in a virtual reality (VR) environment where visuomotor feedback is coupled and with either no reward (*no reward VR*) or a learning task with water reward (*goal-directed VR*). (C) Example two-photon imaging field of view (left) with arrows indicating two example neurons that displayed orientation-selective response for either the stimulus that was repetitively presented (R) (middle, orange) or for the grating orthogonal to the repetitive grating (O_R_) (right, gray). Responses (ΔF/F_0_) of the example neurons to the 4 oriented gratings for each trial are shown in gray with average response across trials in black (A, angled; O_A_ orthogonal to A). Tuning curves show the peak response at the preferred orientation. Scale bar: 50 μm. (D) Change in the proportion of selective neurons from pre to post day for the R and O_R_ gratings (*no stimulus*: p = 0.918; *passive viewing*: p = 0.030; *no reward VR*: p = 0.199; *goal-directed VR*: p < 0.001; Student’s t test). (E) Relative change from pre to post day between the R and O_R_ gratings (RO_index_; left; p = 0.001; one-way ANOVA with LSD test) and other control orientations (O_A_ and O_R_; O_index_; right; p = 0.633; one-way ANOVA). (F) Shift in the orientation selectivity at the population level from pre to post day. Difference in the average maximal response vector across all neurons for each animal is shown as the mean across animals for each group. Bars above the red dashed line indicate a shift toward the R grating, and bars below the dashed line indicate a shift away from the R grating (effect shown in schematic tuning curves on left; p < 0.001; one-way ANOVA with LSD test). (G) Average change in the decoding accuracy across animals from pre to post day for each orientation (left) using a Bayesian maximum-likelihood decoder to determine individual neuronal response to each oriented grating, averaged across the population for each animal. Average change in decoding accuracy for the R grating across animals for each group is shown (right; p < 0.001; one-way ANOVA with LSD test). All panels: ^∗^p < 0.05, ^∗∗^p < 0.01, ^∗∗∗^p < 0.001; n = 9 mice for each group; error bars: SEM. See also Figures S1 and S2 and Table S2.

We characterized the visual responses of layer 2/3 neurons expressing the genetically encoded calcium indicator GCaMP6 [37] in V1 by using two-photon calcium imaging in head-fixed mice that were freely running on a cylindrical treadmill (Figure 1). We first established the baseline level of orientation selectivity in individual neurons on an initial testing day (referred to as “pre”) by presenting a series of full-field oriented gratings (four orientations; single-screen, contralateral to imaged V1). Animals were then presented with only a single oriented grating for 5 consecutive days (repetitive stimulus presentation; 15-min session per day), followed by a second assessment of visual responses to all oriented gratings at the end of the experiments (referred to as “post”; Figure 1A). In a control group, no grating was presented during the 5 consecutive days between pre and post imaging (*no stimulus* group; Figure 1B).

During the presentation of the repetitive stimulus over 5 days, animals were divided into experimental groups in order to systematically assess the impact of stimulus-reward association and visuomotor coupling under conditions ranging from a non-rewarded, uncoupled stimulus to a stimulus-reward association learned in a virtual reality (VR) environment (Figure 1B). The first, *passive viewing*, group viewed the single oriented grating in the same experimental conditions as in the pre/post testing days (single screen, same spatial frequency, and moving at a constant temporal frequency), and no reward was given. Therefore, this condition is representative of traditional laboratory settings used to probe visual responses of neurons in V1 with oriented gratings on a single screen (Figure 1B). The second, *no reward VR*, group represents a more naturalistic condition in a virtual environment, consisting of a linear corridor with the repetitive grating presented on the corridor walls and the optic flow of the stimulus directly linked to the animals’ movements on the treadmill, creating coupled visuomotor feedback. Animals passively viewed the repetitive stimulus over consecutive days with no reward and no requirement to be directly behaviorally engaged. The third, *goal-directed VR*, group viewed the repetitive stimulus while performing a rewarded learning task that required direct behavioral engagement. This later group also had coupled visuomotor feedback in the virtual environment and, additionally, a reward associated to this visual context (Figure 1B). Mice were actively engaged in the task and learned to associate a water reward with the visual context of the virtual corridor (Figure S1). For each animal, the same population of layer 2/3 V1 neurons was imaged across all experimental days (pre, 5 days of repetitive stimulus presentation, and post; see Table S1) and neuronal responses were quantified by the change in mean fluorescence of GCaMP6 (ΔF/F_0_; for example, see Figure 1C).

### Enhanced or Attenuated Stimulus-Specific Representation in V1 Layer 2/3 Neurons

To assess the effect of the repetitively presented stimulus, we first compared visual responses before (pre) and after (post) the 5 consecutive days of repetitive stimulus presentation. We quantified the proportion of neurons that were orientation selective for the repetitively presented grating (R_select_) compared to neurons that were selective for the grating that was orthogonally oriented to the repetitive grating (O_select_; Figure 1C; see STAR Methods). The orthogonal grating was used as an internal control because the animals only saw this oriented stimulus on the pre and post testing days, but not during the 5 days in between. Because animals ran the same amount of time for all gratings on the pre and post days, we included all collected data, regardless of locomotor activity (see also Figure S3).

For the *passive viewing* group, the proportion of R_select_ neurons significantly decreased on the post day compared to pre day (p = 0.004; n = 9; Wilcoxon signed rank), although there was no change in this proportion for the *no reward VR* group (p = 0.301; n = 9; Wilcoxon signed rank) or the group that was not exposed to the repetitive stimulus (*no stimulus* group; p = 0.641; n = 9; Wilcoxon signed rank; Figure S2). In contrast, we found that, by the post testing day, the proportion of R_select_ neurons increased significantly in the *goal-directed VR* group (p = 0.004; n = 9; Wilcoxon signed rank; Figure S2). These changes were specific to the repetitive grating, because we found no significant change in the proportion of O_select_ neurons between pre and post days for any group (*no stimulus*, p = 0.426; *passive viewing*, p = 0.844; *no reward VR*, p = 0.742; *goal-directed VR*, p = 0.078; n = 9 for each; Wilcoxon signed rank; Figure S2). This resulted in a statistically significant interaction between the orientation and experimental group (p < 0.001; two-way analysis of variance [ANOVA]) and a significant change in the proportion of R_select_ compared to O_select_ neurons in the *goal-directed VR* group (Figure 1D; p < 0.001; n = 9; Student’s t test), as well as the *passive viewing* group (Figure 1D; p = 0.030; n = 9; Student’s t test). The stimulus specificity was confirmed by quantifying the relative change of R_select_ compared to O_select_ neurons (RO_index_ = R_select_ − O_select_/R_select_ + O_select_) from pre to post day (Figure 1E). The change in RO_index_ was significantly higher for the *goal-directed VR* group compared to all other groups (Figure 1E; p = 0.001; one-way ANOVA). There was no significant difference across groups when we calculated a similar index between two oriented gratings that mice were only exposed to on pre and post days (Figure 1E; O_index_; p = 0.633; one-way ANOVA). Finally, for the goal-directed task in the VR environment, we found a significant correlation between the proportion of R_select_ neurons and behavioral performance (Figure S1C; R = 0.627; p = 0.005; Pearson’s coefficient).

We then examined how these changes in the proportion of selective neurons affected information encoding within the V1 neuronal population, i.e., the ability to decode, at the population-level, stimulus-specific information based on individual neuronal activity [1]. First, we determined the peak angle of the tuning curve (preferred orientation response vector; see STAR Methods) across neurons for each animal and examined the shift in this vector from pre to post testing days across groups. Although the *no reward VR* and the *no stimulus* groups showed no significant net change in the population orientation vector (0.32° ± 3.37° and 0.42° ± 2.59°, respectively), we found a general shift away from the repetitive stimulus for the passive viewing group (−5.64° ± 1.90°). In contrast, the *goal-directed VR* group showed a shift toward the repetitive orientation (13.81° ± 2.93°), which was significant when compared to all other experimental groups (Figure 1F; p < 0.001; one-way ANOVA). In addition, using a Bayesian maximum-likelihood decoder (see STAR Methods) [1], we found that the average decoding accuracy for the repetitive grating in the *goal-directed VR* group increased from pre to post testing days (10% ± 3%), leading to a significant difference in decoding accuracy in comparison to all other groups (Figure 1G; p < 0.001; one-way ANOVA) but no significant difference in decoding accuracy for the orthogonal grating (p = 0.823; one-way ANOVA).

Therefore, under passive viewing conditions, exposure to a repetitive stimulus at a fixed temporal frequency decreased the proportion of neurons that showed selective responses for that stimulus. Exposure to the same repetitive stimulus in a VR environment (*no reward VR*) resulted in no significant change although the goal-directed task in a virtual environment resulted in enhanced representation of the repetitively presented stimulus.

### Stimulus-Specific Enhanced Representation following Reward Association

**Figure 2:**
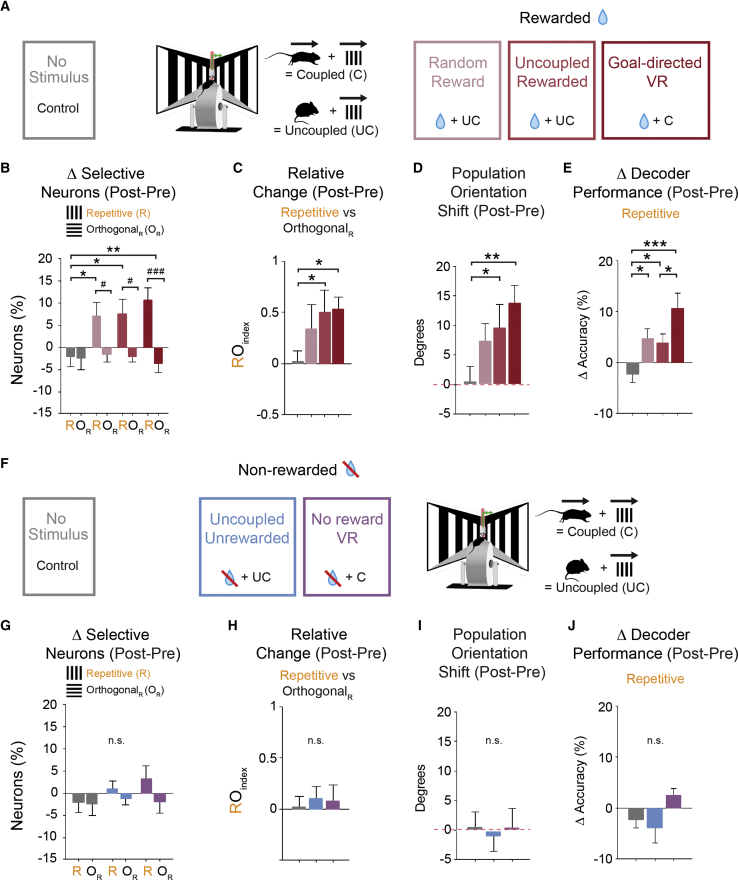
Stimulus-Specific Enhanced Representation following Reward Association (A) Rewarded experimental groups. Over the 5 days of repeated exposure to the oriented grating, mice received a water reward either randomly (*random reward*) or during a learning task within the specified reward zone (*uncoupled*-*rewarded* and *goal-directed VR*). The same visual stimulus was displayed in a virtual environment either uncoupled (*random reward* and *uncoupled-rewarded*) or coupled to the mice locomotion (*goal-directed VR*). The group with no stimulus presentation over 5 days was used as a control. (B) Change in the proportion of selective neurons from pre to post day compared between the R and O_R_ gratings within groups (#p < 0.05; ###p < 0.001; *goal-directed VR*: p < 0.001; *uncoupled-rewarded*: p = 0.013; *random reward*: p = 0.025; Student’s t test) and the change in proportion of R selective neurons compared across groups (p = 0.023; one-way ANOVA with LSD test; ^∗^p < 0.05; ^∗∗^p < 0.01). (C) Relative change from pre to post day between the R and O_R_ gratings (RO_index_; p = 0.032; one-way ANOVA with LSD test; ^∗^p < 0.05). (D) Shift in the orientation selectivity at the population level from pre to post day. Difference in the average maximal response vector across all neurons for each animal is shown as the mean across animals for each group (n = 9 mice for each). Bars above the red dashed line indicate a shift toward the R grating, and bars below the dashed line indicate a shift away from the R grating (p = 0.039; one-way ANOVA with LSD test; ^∗^p < 0.05; ^∗∗^p < 0.01). (E) Change in the decoding accuracy across groups for the R grating from pre to post day using a Bayesian maximum-likelihood decoder to determine individual neuronal response and averaged across the population for each animal (p = 0.003; one-way ANOVA with LSD test; ^∗^p < 0.05; ^∗∗∗^p < 0.001). (F) Non-rewarded experimental groups. Over the 5 days of repeated exposure to the oriented grating, visual stimulus was displayed in a virtual environment either uncoupled (*uncoupled-unrewarded*) or coupled (*no reward VR*), with all groups receiving no reward. The group with no stimulus presentation over 5 days was used as a control. (G) Change in the proportion of selective neurons from pre to post day compared between the R and O_R_ gratings within groups was non-significant for all groups (*no stimulus*: p = 0.641; *uncoupled-unrewarded*: p = 0.322; *no reward VR*: p = 0.301; Student’s t test) and the change in proportion of R selective neurons not significant across groups (p = 0.310; one-way ANOVA). (H) Relative change from pre to post day between the R and O_R_ gratings (RO_index_) was non-significant across non-rewarded groups (p = 0.895; one-way ANOVA). (I) Shift in the orientation selectivity at the population level from pre to post day was non-significant across non-rewarded groups (p = 0.921; one-way ANOVA). (J) Change in the decoding accuracy for the R grating from pre to post day was non-significant across non-rewarded groups (p = 0.123; one-way ANOVA). All panels: n = 9 mice for each group; error bars: SEM. See also Figure S3 and Table S2.

To determine the specific impact of reward and visuomotor coupling on the experience-dependent enhanced representation observed during the *goal-directed VR* task, we first decoupled the stimulus presentation from the motor activity of the mouse (i.e., a visuomotor uncoupled VR playback condition; see STAR Methods); however, the behavioural task remained the same: to lick at the reward zone in order to receive the reward (*uncoupled-rewarded* group; Figure 2A). One consequence of decoupling the visuomotor feedback in this group was that, although animals still performed the task (D5 success rate = 74% ± 11%), their licking was not confined to the immediate reward zone region; hence, the spatial modulation index (SMI) (see STAR Methods) did not increase from day 1 to day 5 (p = 0.652; n = 9; Wilcoxon signed rank). Under these conditions, there was a significant increase in the proportion of R_select_ compared to O_select_ neurons from pre to post testing days (Figure 2B; p = 0.013; n = 9; Student’s t test) and difference in the proportion of R_select_ neurons across groups, such that there was a significant increase compared to the *no stimulus* control but no difference compared to the previous *goal-directed VR* group with coupled visuomotor feedback (Figure 2B; p = 0.023; one-way ANOVA with least significant difference [LSD] test; p = 0.025 *uncoupled-rewarded* versus *no stimulus*; p = 0.440 *uncoupled-rewarded* versus *goal-directed VR*; Table S2).

Likewise, there was a significant increase in both the RO_index_ and shift of the response vector toward the repetitive grating when compared to the *no stimulus* control but no significant difference between this group and the *goal-directed VR* group (RO_index_: Figure 2C; p = 0.032, one-way ANOVA with LSD test; p = 0.019 *uncoupled-rewarded* versus *no stimulus*; p = 0.748 *uncoupled-rewarded* versus *goal-directed VR*; orientation shift: Figure 2D; p = 0.039, one-way ANOVA with LSD test; p = 0.049 *uncoupled-rewarded* versus *no stimulus*; p = 0.350 *uncoupled-rewarded* versus *goal-directed VR*). This increased response to the repetitive stimulus led to an increase in the decoding accuracy from pre to post testing days for the *uncoupled-rewarded* group (p = 0.048; n = 9; paired t test); however, the magnitude of this increase was smaller than the *goal-directed VR* group but still significant when compared to the *no stimulus* control (Figure 2E; p = 0.003, one-way ANOVA with LSD test; p = 0.021 *uncoupled-rewarded* versus *no stimulus*; p = 0.043 *uncoupled-rewarded* versus *goal-directed VR*).

To further confirm the importance of the reward separately from engagement in learning the task, we tested an additional experimental group, *random reward*, in which mice did not have to lick in a designated reward zone but received rewards randomly anywhere along the VR corridor with the same repetitive stimulus presentation as for the *uncoupled-rewarded* group. Here, we also found an increase in the proportion of R_select_ compared to O_select_ neurons from pre to post testing days (Figure 2B) and no significant difference between the *random reward* and the other rewarded groups for the RO_index_, orientation shift toward the repetitive grating, and increase in decoder accuracy for the repetitive grating (Figures 2C–2E). Therefore, the association of the repetitive stimulus with a reward resulted in a stimulus-specific enhanced representation in V1, both after animals had been engaged in learning a rewarded task and when the animals passively received a reward given randomly during stimulus presentation; the rewarded learning task was associated with the largest enhancement on average.

Finally, we removed the reward entirely from the task so that mice were exposed to the same repetitive stimulus in the VR environment with uncoupled visuomotor feedback but received no reward during the 5 consecutive days (*uncoupled-unrewarded* group; Figure 2F). We found no increase in the proportion of R_select_ compared to O_select_ neurons from pre to post testing days (Figure 2G) and no difference across other non-rewarded groups (*no stimulus* control as well as the *no reward VR* group) with regard to the proportion of R_select_ neurons (Figure 2G), RO_index_ (Figure 2H), orientation shift (Figure 2I), or decoding accuracy (Figure 2J). Therefore, in the absence of a reward-associated stimulus, we found either no change of stimulus representation in groups with a dynamic optic flow (either visuomotor coupled [*no reward VR* group] or uncoupled [*uncoupled-rewarded* group]) or a decrease in stimulus representation with a fixed temporal frequency repetitive stimulus (*passive viewing* group; see Figure 1).

### Impact of Locomotion on Experience-Dependent Changes of Stimulus Representation in V1

Because locomotion is known to modulate the gain of visual responses in V1 [21, 24, 27, 33], we assessed whether changes in the proportion of responsive neurons correlated with running behavior. For the rewarded groups, mice were motivated by using water restriction and we found that they spent more time running (total time running, averaged across all days: rewarded 80% ± 2% versus non-rewarded 34% ± 1%; see Figure S3A). However, the difference in the amount of running from pre to post days was highly variable across animals, even within groups (Figure S3B). We found no significant correlation between the change in the proportion of R_select_ neurons and the proportion of time spent running either during pre and post days (Figure S3C; R = 0.206; p = 0.103; Pearson’s coefficient) or across all experimental days (Figure S3D) within any of the experimental groups, i.e., for any given experimental condition, the animals that ran more did not have larger changes in the proportion of R_select_ neurons. Although there was a positive correlation in this regard across all conditions (Figure S3D; R = 0.445; p < 0.001; n = 63; Pearson’s coefficient), this became non-significant when we analyzed, separately, the groups that were rewarded (R = −0.045; p = 0.823; n = 27; Pearson’s coefficient) versus non-rewarded (including control; R = 0.068; p = 0.695; n = 36; Pearson’s coefficient), again, suggesting that the presence of the reward was the driving factor.

Importantly, on the pre and post testing days, there was no bias for the animals to run more during the repetitive grating compared to other orientations and no change in the modulation of responses by locomotion from pre to post days (Figures S3E–S3G). Together, this suggests that locomotion did not directly affect the change in visual response properties on the pre and post imaging days within each experimental group. However, because the presence of the reward itself increased engagement and, subsequently, running time was higher for all rewarded groups within the 5 days of stimulus exposure, it is possible that running induced or enhanced plasticity mechanisms associated with the stimulus-reward association.

### Stimulus-Reward Associations Enhance Selectivity and Response Reliability

**Figure 3:**
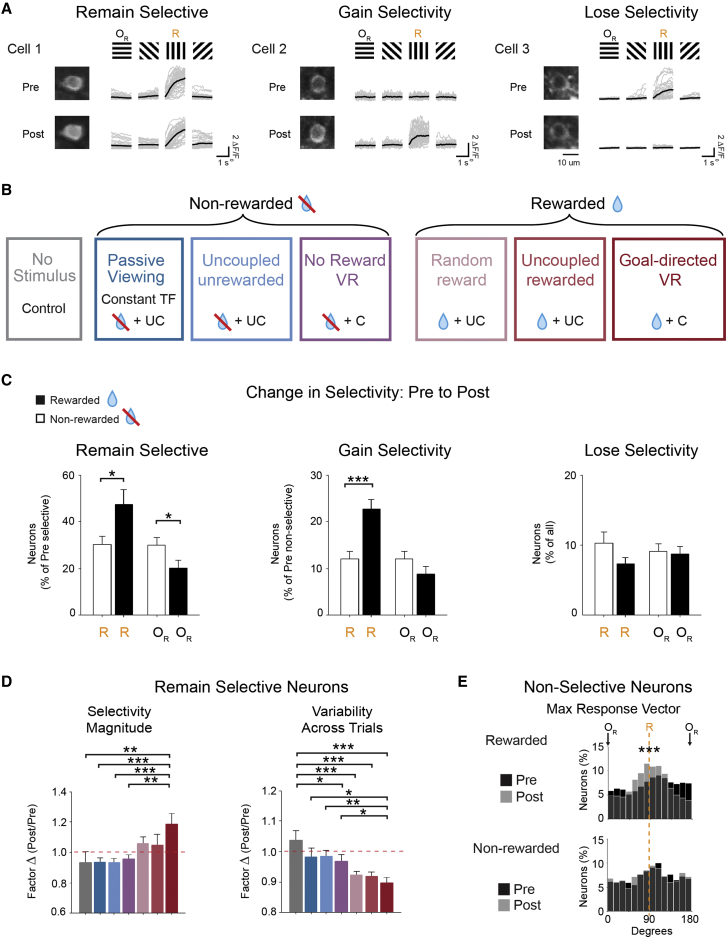
Stimulus-Reward Association Increases Selectivity and Decreases Trial-by-Trial Variability (A) Responses (ΔF/F_0_) to the four oriented gratings for three example neurons before (pre) and after (post) 5 days of repetitive stimulus presentation are shown for neurons that either remain selective (left), gain selectivity (middle), or lose selectivity (right) for the stimulus that was repetitively presented (R). Individual trials are shown in gray and average response across trials in black. (B) Experimental groups depending on reward association and visuomotor coupling during the repetitive visual stimulus over 5 days. Rewarded and non-rewarded groups are indicated. Visuomotor feedback was either coupled (C) or uncoupled (UC). (C) Proportion of the total number of neurons that are selective for the R grating or the total number of neurons that are selective for the O_R_ grating on the pre testing day for the rewarded (black) and non-rewarded (white) groups that then either remain selective (left; R, p = 0.016; O_R_, p = 0.0421) or gain selectivity (middle; R, p < 0.001; O_R_, p = 0.144) by the post testing day. Proportion of the total number of all neurons in the rewarded (black) and non-rewarded (white) groups that then lose (right; R, p = 0.093; O_R_, p = 0.816) selectivity for the R or the O_R_ grating is shown. For all, n = 27 mice; Student’s t test. (D) Factor change in the magnitude of selectivity (preferred orientation response vector; left; p = 0.006, one-way ANOVA with LSD test; ^∗∗^p < 0.01; ^∗∗∗^p < 0.001) and in the variability of the selectivity across trials (as measured by the coefficient of variation; right; p = 0.004, one-way ANOVA with LSD test; ^∗^p < 0.05; ^∗∗^p < 0.01; ^∗∗∗^p < 0.001) on post day compared to pre day (post/pre) for the population of neurons that remain selective for the R grating for each experimental group in (B). Red dashed line represents no change from pre to post. (E) Distribution of the preferred orientation (maximum response vector in degrees) for the individual neurons that remain non-selective from pre (black) to post (gray) testing day for rewarded (^∗∗∗^p < 0.001; Kolmogorov-Smirnov test) and non-rewarded (p = 0.914; Kolmogorov-Smirnov test) groups. For each condition, both distributions are overlaid to show the changes between pre and post days. Dashed line represents the orientation of the R grating. All panels, error bars: SEM. See also Table S2.

We next investigated the single-cell dynamics underlying experience-dependent changes by following the selective properties of each individual neuron across pre and post days (Figure 3). We grouped neurons into four categories: a neuron selective for the repetitive grating on the initial pre day can either “remain selective” or “lose selectivity” on the post day; alternatively, neurons that are not initially selective for the repetitive grating on the pre day can either “gain selectivity” or remain “non-selective” by the post day (Figure 3A). The increased proportion of R_select_ neurons in the rewarded groups (Figure 3B) resulted from both more neurons that remained selective (Figure 3C; p = 0.016; n = 27; Student’s t test) as well as more initially non-selective neurons that gained selectivity (Figure 3C; p < 0.001; n = 27; Student’s t test). For the population of stable R_select_ neurons (i.e., remain selective neurons), the selectivity magnitude increased for the *goal-directed VR* group in comparison to the *no stimulus* and all non-rewarded conditions, and the variability across trials (coefficient of variation) was significantly lower for the rewarded groups compared to the *no stimulus* control (Figure 3D; Table S2).

Lastly, there was a significant shift in the distribution of the maximum response vector toward the repetitive stimulus within the population of neurons that remained non-selective from pre to post testing days for the rewarded groups and no significant change for the non-rewarded groups (Figure 3E; rewarded: p < 0.001; non-rewarded: p = 0.914; Kolmogorov-Smirnov test), similar to that observed at the level of the whole population (see Figure 2). Therefore, reward association with the repetitive stimulus resulted in more neurons showing stable, more reliable, and more selective responses to this stimulus, leading to a globally enhanced stimulus representation in both selective and non-selective neurons.

### Enhanced Representation of Two Equally Rewarded Stimuli and Increased Discriminability

**Figure 4:**
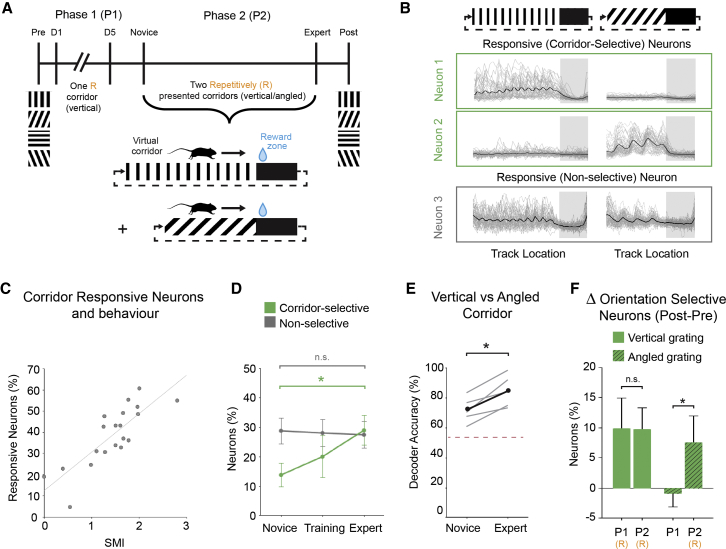
Enhanced Representations of Two Repetitively Presented and Equally Rewarded Stimuli (A) Experimental timeline. Orientation selectivity was assessed on the day before (pre) phase 1 (P1) training with a single virtual corridor (vertical gratings) followed by phase 2 (P2), where two virtual corridors were repetitively presented with a water reward, and a final assessment of orientation selectivity (post). (B) Responses (ΔF/F_0_) of 3 example neurons; individual trials (gray) and average response (black). Neuron 1 and 2: corridor-selective responses are shown. Neuron 3: corridor responsive neuron is shown, not selective for a single corridor grating but responsive to both corridors. (C) Correlation between the percentage of corridor-responsive neurons and the behavioral performance (spatial modulation index [SMI]; R = 0.82; p < 0.001; n = 15 [novice, mid-training, and expert sessions from 5 mice]; Pearson’s coefficient). (D) Proportion of corridor-specific (green) and corridor-responsive (gray) neurons on the first day of phase 2 (novice), the midpoint training day (i.e., after first half of training days; training), and the last day of training (expert). Increase in the percentage of corridor-selective neurons from novice to expert day is shown (p = 0.047; n = 5; Wilcoxon signed rank). (E) Accuracy of a template-matching decoder to determine which corridor grating (vertical or angled) was presented per trial for the novice and expert day (p = 0.031; n = 5; Wilcoxon signed rank). Dashed line represents chance level. (F) Change in the proportion of orientation selective neurons from pre to post day for both vertical gratings (p = 0.905; Mann-Whitney U test) and angled gratings (p = 0.032; Mann-Whitney U test) for animals that only had phase 1 (P1; 4 mice) and animals that had both phase 1 and phase 2 (P2; 5 mice). Repetitively presented stimuli are indicated (R). All panels, error bars: SEM.

Because we found that the representation of a reward-associated stimulus was enhanced in V1, we next tested how an additional rewarded stimulus would be represented: whether the second stimulus would be comparatively enhanced and whether the discriminability between these two equally rewarded stimuli would be maintained or increased. To this end, after the first phase of *goal-directed VR* learning with one repetitive stimulus, 5 mice were used for a second phase, where they were presented with an additional grating along the virtual corridor (Figure 4A). Here, the length of the corridors was different between the two gratings (vertical and angled) to encourage the separation of these virtual environments as different contexts, but all other task parameters remained the same. We found that a population of layer 2/3 neurons responded to the visual stimulus presentation along the virtual corridors (corridor responsive neurons; Figure 4B): these neurons were defined by having a significantly larger response across trials when the corridor walls contain a visual stimulus compared to black corridor walls (p < 0.001; paired t test). We found a positive correlation between the percentage of corridor responsive neurons and the behavioral performance (SMI; Figure 4C; R = 0.82; p < 0.001; n = 15 [novice, mid-training, and expert sessions from 5 mice]; Pearson’s coefficient), indicating that the proportion of corridor responsive neurons in V1 is related to task engagement.

The corridor responsive V1 population consisted of neurons that were either responsive to both corridors (e.g., neuron 3 in Figure 4B) or selectively responsive to only one of the corridor gratings (corridor-selective neurons; e.g., neurons 1 and 2 in Figure 4B). Only the proportion of the corridor-selective neurons significantly increased from the novice to the expert days (Figure 4D; novice: 13% ± 4%; expert: 29% ± 5%; p = 0.047; n = 5; Wilcoxon signed rank). Because there were significantly more neurons that responded to only one corridor, decoder accuracy (i.e., population-level stimulus-discriminability using a template-matching decoder) between these two corridors significantly increased from the novice to expert day (Figure 4E; pre: 71% ± 4%; post: 84% ± 5%; p = 0.031; n = 5; Wilcoxon signed rank). Therefore, the increase in responses to rewarded stimuli in V1 is also context specific and results in, not only a general increase in the proportion of responsive neurons, but also an increase in the level of stimulus discriminability between stimuli of similar relevance in V1 neuronal population.

Finally, we compared the orientation selectivity of neurons for mice from phase 1 and phase 2. For animals that received phase 2 training with both vertical and angled repetitive gratings, we found an increased proportion of neurons responded to the angled grating on the post testing day, although the mice from phase 1 showed no change in the representation of angled gratings (Figure 4F; phase 1: −1% ± 2%; phase 2: 8% ± 4%; p = 0.032; Mann-Whitney U test). Interestingly, we found that there was no additive increase in the response to vertical gratings from phase 1 to phase 2 (Figure 4F; phase 1: 10% ± 5%; phase 2: 10% ± 4%; p = 0.905; Mann-Whitney U test), indicating that the experience-dependent changes that occur after 5 days of exposure to a repetitive stimulus are maintained, but not amplified further by additional days of exposure to the same stimulus.

### Different Subpopulations of Grating-Selective Neurons across Viewing Contexts

**Figure 5:**
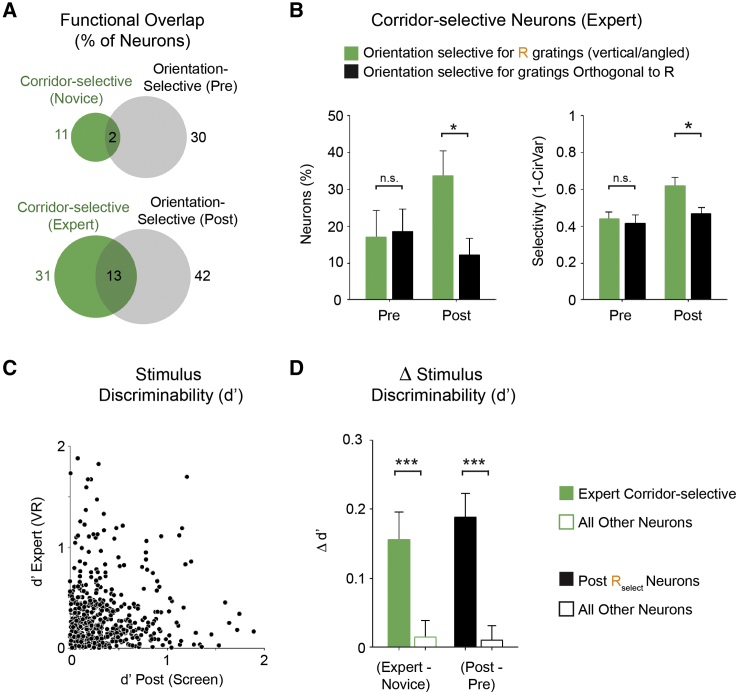
Different Subpopulations of Grating-Selective Neurons across Viewing Contexts (A) Percentage of corridor-selective neurons in the virtual environment (green; for novice [top] or expert [bottom]) and orientation-selective neurons during single-screen viewing (gray; selective for any orientation [either 0°, 45°, 90°, or 135°] on pre [top] or post [bottom] testing day); percentage of overlapping indicated in center. (B) Among the population of corridor-selective neurons on expert day: the proportion that is orientation selective for the R gratings (green; either vertical or angled) or for the gratings orthogonal to the R gratings (black) is shown on the left and, on the right, the magnitude of their selectivity for both pre and post testing days. ^∗^p < 0.05; n = 5 mice; Mann-Whitney U test. (C) Stimulus discriminability (d’) between vertical and angled gratings in the VR on expert day and between the vertical and angled gratings under single-screen viewing conditions on post testing day for each neuron (n = 510 neurons from 5 mice). (D) Change in d’ from novice to expert day between the two R gratings in the virtual environment (vertical and angled) for the corridor-selective population (green; n = 158) versus all other neurons (n = 352) and change in d’ from the pre to post testing day during single-screen viewing for the population of neurons that are orientation selective for the R grating (R_select_; black; n = 166) versus all other neurons (n = 344). ^∗∗∗^p < 0.001; Mann-Whitney U test. All panels, error bars: SEM.

We then investigated the overlap between V1 neurons that showed grating-selective properties in different environmental contexts (i.e., different visual display): either in the virtual environment (corridor-selective neurons) or when viewing gratings of the same orientation with monocular stimulation on a single screen (orientation selective neurons on pre and post testing days). Before training, we found that largely distinct populations were selective under the two conditions, with only a minority of overlap (Figure 5A; 2% of the total population); after training in the virtual environment, the proportion of overlap increased but remained in the minority (Figure 5A; 13% of the total population). Specifically, among the corridor-selective population on expert day, we found that an equal proportion of neurons were orientation selective for the repetitive gratings (i.e., vertical/angled; 17% ± 7%) and orthogonal gratings (18% ± 6%) on the pre day (p = 0.955; n = 5; Mann-Whitney U test) and that the magnitude of their selectivity was also equal (vertical/angled: 0.44 ± 0.04; orthogonal: 0.42 ± 0.05; p = 0.328; n = 5; Mann-Whitney U test; Figure 5B). By the post day, the proportion of the corridor-selective population that was orientation selective for vertical/angled gratings (33% ± 7%) increased in comparison to the orthogonal gratings (12% ± 4%; p = 0.022; n = 5; Mann-Whitney U test), as did the selectivity magnitude (vertical/angled: 0.62 ± 0.04; orthogonal: 0.46 ± 0.03; p = 0.006, respectively; n = 5; Mann-Whitney U test; Figure 5B). However, even after learning, only a minority of the population that was selective for a specific corridor in the VR environment was selective for that same grating during single-screen viewing.

To examine the ability for an individual neuron in V1 to distinguish between the two behaviorally relevant gratings (vertical/angled) under these different viewing contexts, we used a measure of stimulus discriminability (d’; see STAR Methods) between vertical and angled gratings. We found that stimulus discriminability across contexts was not correlated (R = 0.079; p = 0.077; n = 510 neurons; Pearson’s coefficient): most neurons showed discriminability in either the VR environment or during single-screen viewing, but not under both conditions (Figure 5C). However, the discriminability between the two repetitive gratings increased with training for both the corridor-selective population (change in d’ from novice to expert day in the VR environment; p < 0.001; Mann-Whitney U test) and the R_select_ population of neurons (change in d’ from pre to post testing day during single screen presentation; p < 0.001; Mann-Whitney U test; Figure 5D). This suggests that there is a mechanism for the transfer, or generalization, of stimulus-specific information during training in the VR environment to the population of R_select_ neurons in a different environmental context on the post testing day.

### Generalization of Reward-Associated Responses

**Figure 6:**
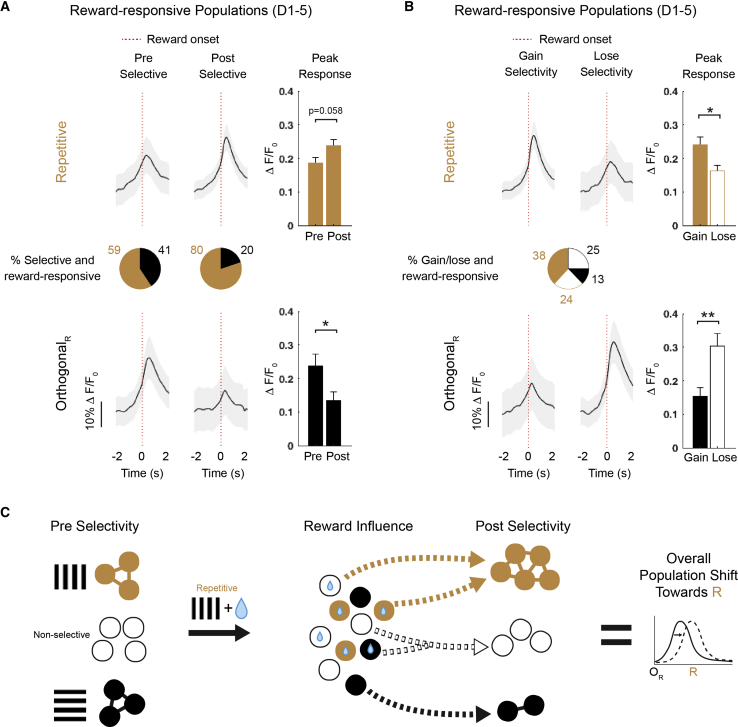
Generalization of Reward-Associated Activity across V1 Subpopulations and Viewing Contexts (A) Average activity (ΔF/F_0_) across trials for all animals that received rewards; activity associated with reward onset (time = 0; ±2-s window) is shown as traces, averaged over neurons in selected populations: reward-responsive and selective for the R grating (orange) on either pre (n = 108) or post (n = 150) testing days (top) or reward responsive and selective for the orthogonal to the R grating (orthogonal_R_; black) on either pre (n = 74) or post (n = 37) testing days (bottom). Proportion of total for these populations is shown as pie charts for pre and post days. Average peak response (max ΔF/F_0_ within window) for these populations is shown on right (repetitive selective: p = 0.058; orthogonal selective: p = 0.023; Student’s t test). (B) Average activity (ΔF/F_0_) across trials for all animals that received rewards; activity associated with reward onset (time = 0; ±2-s window) is shown as traces, averaged over neurons in selected populations: reward-responsive and gained selectivity (n = 113; solid orange) or lost selectivity (n = 71; open orange) for the R grating on post testing day (top) or reward responsive and gained selectivity (n = 38; solid black) or lost selectivity (n = 74; open black) for the orthogonal_R_ on post testing day (bottom). Proportion of total for these populations is shown as pie chart. Average peak response (max ΔF/F_0_ within window) for these populations is shown on right (repetitive: p = 0.010; orthogonal selective: p = 0.001; Student’s t test). (C) Summary schematic of conditions underlying generalization of reward-associated activity across environmental contexts and days. Populations selective for the R grating (orange), orthogonal_R_ (black), and non-selective (white) are indicated. Stimulus-associated reward influences shift V1 populations responses toward the R grating on the post testing day. All panels: ^∗^p < 0.05; ^∗∗^p < 0.01; ^∗∗∗^p < 0.001; error bars: SEM.

We next investigated the potential mechanisms for the generalization of stimulus-specific reward association across environments. We examined reward influences on neuronal responses in all experimental animals that received rewards. We first defined reward-responsive neurons during D1–D5 as having significantly higher activity in a 1-s window following the reward onset compared to a 1-s window preceding the reward onset (Figure 6A; see also STAR Methods). We then examined the properties of orientation-selective neurons that were also reward responsive during D1–D5 of the repetitive presentation. We found that the proportion of reward-responsive R_select_ neurons compared to O_select_ neurons was higher on the post day (i.e., after exposure to the reward-associated repetitive stimulus; 80% versus 20%, respectively; Figure 6A). Additionally, although the peak response to the reward onset for the R_select_ neurons tended to increase from the pre to post selective population, reward-responsive post-O_select_ neurons had a significantly lower peak response to the reward onset (Figure 6A; p = 0.023; Student’s t test). These results suggest that the population of post-R_select_ neurons had stronger reward influences during the repetitive grating sessions on D1–D5 compared to the population of post-O_select_ neurons.

Indeed, when we then examined the response properties of neurons that were reward-responsive during D1–D5 and either gained or lost selectivity from the pre to post days, we found that a higher proportion of neurons that *became* R_select_ had previously been reward responsive during D1–D5 and that these neurons that gained selectivity also had significantly higher peak responses to the reward onset (Figure 6B; p = 0.010; Student’s t test). Importantly, the converse was true for O_select_ populations; neurons that *lost* their selectivity from the pre to post day showed significantly higher peak reward responses during D1–D5 (Figure 6B; p = 0.001; Student’s t test). Ultimately, strong reward responses during D1–D5 were associated with a gain in selectivity for the repetitive stimulus, resulting in more R_select_ neurons and a loss of selectivity in O_select_ neurons. As a result, enhanced reward-related responses in the VR context transferred to stimulus-specific responses in the pre/post context. This led to an overall enhancement of the repetitive stimulus representation with a shift of population responses toward the repetitive grating, driven by reward associations (Figure 6C; see also Figures 1F and 2D).

## Discussion

In this study, we demonstrated that the association of a reward with a repetitive visual stimulus over 5 consecutive days resulted in an enhanced representation of the reward-associated stimulus in mouse V1 layer 2/3 neurons, while responses to other stimuli were unaffected. This experience-dependent plasticity was observed both after animals had been engaged in learning a rewarded task and when the reward was given randomly during the stimulus presentation. By following changes in single-cell activity over time, we found that both a gain in selectivity for the rewarded stimulus and an increase in response reliability of previously responsive neurons underlie the net enhancement of the repetitive grating representation with reward. When a second stimulus was paired with a reward, we found a similar enhanced representation of this stimulus as well as increased discriminability between both stimuli of similar reward value. Enhanced responses to rewarded stimuli generalized across V1 subpopulations and viewing contexts. In the absence of reward, there was either no change of stimulus representation over days or a decrease in stimulus representation when the stimulus was consistently viewed at a fixed temporal frequency. Taken together, our findings highlight the importance of the behavioral relevance of sensory stimuli in experience-dependent plasticity in primary sensory cortices to enhance and stabilize the representation of rewarded features while suppressing responses to non-relevant stimuli.

Previous studies investigating changes in V1 after learned associations between a visual stimulus and a reward have also found an enhanced representation of task-relevant stimuli in layer 2/3 neurons [3, 4, 12, 15, 16] and a difference in adaptation between non-rewarded (passive) and rewarded conditions [12]. Our results extend these conclusions by systematically testing the impact of visuomotor coupling, stimulus-reward association, and goal-directed task engagement on this plasticity. We conclude that the main factor leading to enhanced stimulus-specific representation in V1 neurons is the association of the repetitive stimulus with a reward; this enhancement was observed after both coupled and uncoupled visuomotor experience and after both expected (active learning) and unexpected (randomly given) rewards. Beyond an increase in the proportion of neurons responsive to the repetitive stimulus, we also found that stimulus-reward associations led to network-wide plasticity: with shifts in population selectivity and increased information encoding, discriminability, and reliability in responses over time. The rewarded task was a simple detection task, but our results are consistent with previous studies using a more challenging learning discrimination task [3, 15, 38], which also demonstrated that enhanced selectivity of task-relevant stimuli was largely due to the stabilization of existing responses and the recruitment of previously non-responsive neurons. However, in these studies, selectivity is generally defined by the difference in the responses to the rewarded and the non-rewarded stimuli, both of which are modified by experience. This suggests that changes in responses to individual gratings differ for discrimination tasks, where a non-rewarded stimulus can gain relevance by providing a “no-go” signal, compared to a detection task, where non-rewarded stimuli act as irrelevant signals.

In addition to the enhanced encoding of a single rewarded stimulus, we also found similar increased representations of two equally rewarded stimuli and increased discriminability between these rewarded stimuli with learning. This demonstrates that, even if two separate contexts hold the same behavioral relevance (each led to the same probability of a reward), experience-dependent changes in V1 promote not only increased efficiency in stimulus encoding but also discriminability between equally relevant contexts. This highlights the capacity of V1 networks to encode both the value (relevance) of a stimulus as well as its specificity compared to equally valuable stimuli. Furthermore, in line with previous studies, we found that experience-dependent plasticity occurring within the first few days of exposure to a repetitive stimulus is maintained, but not amplified further with additional exposure to the same stimulus, and that plasticity in response to a new repetitive stimulus can be induced concurrently [7]. The representation of the new repetitive stimulus was enhanced to the same extent as the first rewarded stimulus, highlighting specific network constraints for this type of experience-dependent plasticity. Further experiments are required to determine the capacity of the network to maintain more than two enhanced representations of rewarded stimuli simultaneously and to establish the conditions of the potential extinction of these representations [39].

In contrast to our results with stimulus-reward associations, we observed stimulus habituation during passive viewing of a repetitive stimulus at a fixed temporal frequency. These findings potentially contrast with experiments showing stimulus-specific response potentiation to a repetitive stimulus under similar conditions, using chronic electrophysiological recordings of visually evoked potentials in layer 4 [7, 8, 9] (but see [40]). Our results are in agreement with a previous two-photon calcium imaging study that observed a stimulus-specific decrease in the proportion and selectivity of layer 2/3 neurons after passive viewing of a grating [5]. Although we found stimulus-specific habituation when the stimulus was displayed at a fixed temporal frequency and uncoupled to the animals’ movements, no change was observed when the same stimulus was not predictable (uncoupled playback) or when it was coupled to the animal’s locomotion, mimicking natural optic flow. This is consistent with the idea that habituation occurs for stimuli that are learned to be irrelevant for the animal’s behavior [11].

Our results revealed that the same oriented gratings elicited responses in largely separate neuronal populations whether displayed in the virtual environment or viewed under visual stimulation conditions with a single screen [41]. Although pairing of the reward with an oriented grating occurred only when animals were in the virtual environment, the enhanced representation occurred in both subpopulations of neurons, i.e., generalized across neurons that were not observed to respond selectively during pairing with the reward. One hypothesis is that stimulus-related reward associations may strengthen existing connections between excitatory neurons of different orientation preference and shift orientation selectivity within individual neurons [42]. Additionally, some neurons may display a bias in their subthreshold responses during pairing with the reward, which could not be detected with GCaMP6 imaging. Further investigations are needed to characterize how these changes generalize to neurons responding to the same orientation in different contexts and to determine the specific connectivity between these neuronal ensembles. A recent study using a computational model of V1 layer 2/3 neuronal circuits has shown that specialized interneuron circuits could store information about rewarded stimuli and instruct changes in two excitatory networks with, for instance, different visual receptive field locations [17]. This transfer of reward-associated enhanced responses across subpopulations is consistent with the observation of invariance of learned representations, such that stimulus representations are generalized across variable viewing contexts and receptive fields.

As a growing number of studies demonstrate altered levels of neuronal activity according to behavioral-state-dependent changes, in subcortical regions, such as the superior colliculus [43], thalamus [24, 44, 45], and cerebellum [46, 47, 48], as well as contextual and experience-dependent changes in higher cortical areas with reciprocally connections to V1 [49], such as the retrosplenial cortex [50, 51, 52, 53, 54], the anterior cingulate cortex [35, 55], parietal cortex [56], and visual association cortex [41, 57, 58], it seems likely that these experience-dependent changes in V1 involve modulation of activity in circuits at multiple processing levels. Additionally, neuromodulatory inputs likely play an important role in behavioral-state-dependent plasticity, as cholinergic and noradrenergic influence have already been shown to modulate cortical plasticity, attention, and learning [59, 60] in V1, as well as responses to locomotion and behavioral-state changes [22, 25, 61, 62]. Indeed, one potential confounding factor in this study is the time that mice spent running during the presentation of the repetitive grating—because locomotion has been shown to modulate both visual responses and plasticity of V1 layer 2/3 neurons [10, 13, 27, 31, 63, 64]. However, locomotion itself has been shown to increase the gain of visual responses [21], but not directly affect the orientation tuning of individual neurons [21, 30] or general visual acuity [32]. For instance, previous work has found that viewing an oriented grating for 60 min per day while running and without being rewarded led to a shift in orientation toward a repetitive stimulus and sharpening of orientation tuning [10], as well as a general increase in stimulus-specific information and response reliability [31]. These studies suggest that locomotion is required for enhanced stimulus encoding, an idea consistent with literature showing increased experience-dependent plasticity in V1 with running [64]. In the current study, we show that, for shorter daily exposure to a stimulus (15 min per day), the absence of a reward prevented the stimulus response enhancement. However, mice in the unrewarded groups also spent less total time running than mice in the rewarded groups, but there was no significant correlation between the enhanced stimulus-specific representation and the proportion of time spent running within any of the experimental groups, i.e., the animals that ran more did not have larger response changes. Therefore, in the current study, running time did not appear to be the main factor correlated with the magnitude of the observed enhanced stimulus representation in V1. However, because the presence of the reward itself increased levels of motivation and engagement, resulting in increasing total running time, it is possible that running time higher than a certain threshold induces or enhances plasticity of stimulus-evoked responses.

Altogether, our results show that repeated exposure to a visual stimulus leads to enhanced responses when the stimulus was associated with a reward and to either no change or attenuated responses when the stimulus was non-rewarded. This mechanism could prove to be highly adaptive for behavior by suppressing responses to irrelevant stimuli that may act as distractors while optimizing stimulus encoding and information processing for behaviorally relevant stimuli, such as those associated with a reward.

## STAR★Methods

### Key Resources Table

**Table:** 

REAGENT or RESOURCE	SOURCE	IDENTIFIER
**Bacterial and Virus Strains**		

AAV1.Syn.GCaMP6s.WPRE.SV40	Addgene	RRID:Addgene_100843
AAV1.Syn.GCaMP6f.WPRE.SV40	Addgene	RRID:Addgene_100837

**Experimental Models: Organisms/Strains**		

Mouse: C57BL/6J	The Jackson Laboratory	RRID:IMSR_JAX:000664

**Software and Algorithms**		

MATLAB 2013/2017a	Mathworks	RRID:SCR_001622
Psychophysics Toolbox package for MATLAB	http://psychtoolbox.org	RRID:SCR_002881
LabVIEW version 8.2	National Instruments	RRID:SCR_014325
Jet Ball system	PhenoSys	RRID:SCR_004072
ThorImage LS Microscopy Software	Thorlabs	version 2.4
ViRMEn	[65]	https://pni.princeton.edu/pni-software-tools/virmen
SIMA 1.3.2 (sequential image analysis)	[66]	https://pypi.org/project/sima/
ScanImage	Vidrio Technologies	RRID:SCR_014307
FISSA	[67]	https://github.com/rochefort-lab/fissa
ImageJ (Fiji)	NIH – public domain	http://fiji.sc; RRID:SCR_002285
Analyses were performed using custom-written MATLAB scripts	This paper	https://github.com/rochefort-lab

**Other**		

Optical encoder	Pewatron	E7P, 250cpr
Reward spout	Harvard Apparatus	Cat#59-8636
Capacitive touch sensor	Sparkfun	Cat#SEN-12041

### Lead Contact and Materials Availability

Further information and requests for resources and reagents should be directed to and will be fulfilled by the Lead Contact, Janelle MP Pakan (janelle.pakan@med.ovgu.de). This study did not generate new unique reagents.

### Experimental Model and Subject Details

#### Subjects

All animal experiments were approved by either the Animal Welfare and Ethical Review Board (AWERB) of the University of Edinburgh (and experiments performed under a project license granted by the UK Home Office) or by the animal care committee of Sachsen-Anhalt, Germany, and conformed with the UK Animals (Scientific Procedures) Act 1986 and the European Directive 86/609/EEC and 2010/63/EU on the protection of animals used for experimental purposes.

Animals were group housed (typically 2–4 mice) and a total of seven experimental groups were included in the study, each with 9 mice per group (see Table S1). Both male and female mice, aged 6-12 weeks, with a C57BL/6J background were used (RRID: IMSR_JAX:000664; Jackson Laboratory). Mice were housed in standard cages at a 12h/12h light dark cycle, food and water were provided *ad libitum* (except for during behavioral testing involving reward, see below).

### Method Details

#### Surgery

For cranial window implantation and virus injection, mice were anaesthetized with isoflurane (4% for induction and 1%–2% maintenance during surgery) and mounted on a stereotaxic frame (David Kopf Instruments). Eye cream was applied to protect the eyes (Bepanthen, Bayer), analgesics and anti-inflammatory drugs were injected subcutaneously (buprenorphine, 0.1 mg/kg of body weight, carprofen, 0.15mg, and dexamethasone, 2μg). A section of scalp was removed, and the underlying bone was cleaned before a craniotomy (approximately 2x2 mm) was made over the left V1 (centered around 2.5 mm lateral and 0.5 mm anterior to lambda). Then an adeno-associated virus (AAV) was injected using a pipette with ~20 μm tip diameter (Nanoject, Drummond Scientific) at a speed of 10 nL min^-1^ at depths throughout the cortex (to label a cortical column, 2-3 injection sites were made ranging 250-600 um deep; 50-100 nL per injection site for a total volume of 200 nl). Either AAV1.Syn.GCaMP6s.WPRE.SV40 (RRID:Addgene_100843) or AAV1.Syn.GCaMP6f.WPRE.-SV40 (RRID:Addgene_100837) was injected in V1; GCaMP6s was used for all experimental groups except for the 5 mice trained for both phase 1 and phase 2 in *goal-directed VR* group, for which we used GCaMP6f [see Table S1]; note, there were no significant differences in responses to the repetitive grating between the GCaMP6s and GCaMP6f expressing mice for the *goal-directed VR* group in phase 1, so results were pooled, see Figure 4F). After each injection, pipettes were left *in situ* for an additional 5 min to prevent backflow. The craniotomy was then sealed with a glass coverslip and fixed with cyanoacrylate glue (gel control, Loctite). A custom-built head-post was implanted on the exposed skull with glue and cemented with dental acrylic (Paladur, Heraeus Kulzer). After recovery from anesthesia, animals were returned to their home cage for 2-3 weeks to allow for virus expression and clearing of the cranial window [68] before imaging.

#### Two-photon imaging

Two-photon calcium imaging was performed using one of three resonant scanning two-photon microscopes (see Table S1). The first, a custom built 12 kHz resonant scanning system with a Ti:Sapphire pulsing laser (Chameleon Vision-S, Coherent; < 70 fs pulse width, 80 MHz repetition rate) tuned to 920 nm. Images were acquired at 40 Hz (using a 40X 0.8 NA or a 25X 1.05 NA, Nikon objective, see Table S1) with a custom-programmed LabVIEW based software (version 8.2; National Instruments). The second, a 8kHz resonant scanning microscope (B-scope, Thorlabs) with a Ti:Sapphire pulsing laser (Chameleon Ultra II, Coherent; 140 fs pulse width, 80 MHz repetition rate) tuned to 920 nm. Images were acquired at 30 Hz (using a 20X objective 1.0 NA, Olympus) with ThorImageLS software (version 2.4., Thorlabs). The third, using an 8kHz resonant scanning microscope (HyperScope, Scientifica) with an Ultasfast laser (InSight X3 Dual output laser; Spectra-Physics; < 120 fs pulse width, 80 MHz repetition rate) tuned to 940 nm. Images were acquired at 30 Hz (using a 16X objective 0.8 NA, Nikon, zoom factor 2x) with ScanImage software (Vidrio Technologies). With all two-photon systems, chronic imaging of the same L2/3 field-of-view (at cortical depths between 180–280 μm) was performed across consecutive days.

For all groups and all imaging sessions, mice were awake, head restrained, and placed on a cylindrical treadmill (either a 20 cm polystyrene cylinder mounted on a ball-bearing axis with the custom two-photon system [69], or an air-suspended polystyrene 20 cm ball with B-Scope and HyperScope systems, which was fixed on both sides so that mice could run freely only in a linear direction). Movements were monitored using an optical encoder (E7P, 250cpr, Pewatron, with custom two-photon system, sampling frequency 12 kHz; or Jet Ball system, PhenoSys GmbH, with B-Scope and HyperScope systems, sampling frequency 60 Hz;). In all cases, the sampling frequency of the optical encoders were down-sampled to meet the sampling frequency of the imaging.

#### Stimulus presentation: pre and post testing days

For each experimental group, the orientation selectivity of neurons within the selected field-of-view for each animal was assessed before (pre) and after (post) the presentation of a repetitive grating while animals were awake and head-fixed, but free to move at will. Visual stimuli were generated using the Psychophysics Toolbox package [70] for MATLAB (Mathworks) and displayed on a single LCD monitor (51 × 29 cm, Dell) placed 20 cm from the eye contralateral to the cranial window, covering 104° x 72° of the visual field. Visual stimulation (10-20 trials) consisted of stationary full-field gratings for 2-4 s and corresponding moving stimulus for 2-3 s (fixed spatial frequency [0.03-0.05 cpd] and constant temporal frequency [1-1.5 Hz], 4 equally spaced orientations in randomized order, contrast 80%, mean luminance 37 cd/m^2^). Each oriented grating was separated by a gray period (isoluminant, 5 s) and each trial started and ended with a gray screen for 2 s. Spontaneous activity in the dark was assessed by randomly interleaving trials where no visual stimulation was presented (~10 trials, 60-90 s each).

#### Stimulus presentation: repetitive stimulus

For the repetitive stimulation days, a single oriented grating at a fixed spatial frequency was displayed in a 15 minute daily session for 5 consecutive days. The display and reward-association of the repetitive stimulus varied across groups based on: (1) either a constant or dynamic temporal frequency, (2) coupling or uncoupling of the visual stimulus to locomotor behavior (i.e., coupled or uncoupled optic-flow), and (3) the presence or absence of a reward associated with the repetitive stimulus (rewarded or unrewarded; see also Table S1). Spontaneous activity in the dark was collected before the repetitive stimulus presentation on each day. The control *no stimulus* group did not receive any stimulus presentation in the 5 consecutive days between the pre and post testing days.

##### Unrewarded experimental groups

For the *passive viewing* group, a full-field grating was displayed on a single screen as in the pre and post testing days (see above). For 5 consecutive days, a single oriented grating was displayed in a 15 minute daily session with grating presentations of 4 s (at same fixed spatial and constant temporal frequency as pre and post testing days), interleaved by isoluminant gray periods of randomized duration between 5 to 15 s, to simulate the trial by trial nature of the *goal-directed VR* task, see below. The total time of grating stimulation per session was 5 min. Mice were able to voluntarily run on the treadmill, however, running speed and visual stimulus presentation were not coupled as the temporal frequency and spatial frequency of the repetitive grating were fixed and constant throughout.

For the *uncoupled-unrewarded* group, mice were placed in a virtual environment (custom two-photon system) consisting of a linear corridor with the repetitive stimulus (vertical oriented grating) on the corridor walls. Animals ran freely, but visuomotor feedback was uncoupled to optic-flow and varied randomly throughout each session independent of the animals movements (i.e., uncoupled VR playback conditions); therefore, the stimulus temporal frequency was dynamic and randomly varied according to the same range of parameters as in the *goal-directed VR* group (see below; range 0.5-3.5 Hz; average 2 Hz across trials).

For the *no reward VR* group, mice were placed in a virtual environment (PhenoSys GmbH; B-Scope two-photon system) consisting of a linear corridor with the repetitive stimulus (vertical oriented grating) on the corridor walls. Animals ran freely, and visuomotor feedback was coupled with optic-flow throughout each session; therefore, the stimulus temporal frequency was dynamic and controlled by the locomotion of each mouse.

##### Rewarded experimental groups

Mice were put on a 1 ml/day water restriction regime 3 days before training commenced to increase motivation during behavioral training. This regime maintained bodyweight at 85%–90% of their free feeding weight, calculated as the mean of the last 3 days before water restriction. Mice were positioned in front of two angled computer screens forming the VR environment with a reward spout within reach. The reward spout (59-8636; Harvard Apparatus, UK) was fitted with a capacitive touch sensor (SEN-12041; Sparkfun, CO, USA) to detect animal licking behavior. Either PhenoSys software (PhenoSys GmbH; for the HyperScope two-photon system) or the MATLAB based package ViRMEn [65] (for the custom two-photon system) combined with custom written code was used to design and run the presentation of the virtual environment and collect related data. The virtual reality system was updated at a rate of 60 Hz. Imaging and behavioral datafiles were aligned post hoc, where the behavioral datafile was matched to the imaging datafile by down-sampling and interpolating such that the aligned dataset had the same number of frames. Training consisted of daily sessions where a single droplet of water (~5 μl/reward) was dispensed per trial (average trials per session for rewarded groups: D1, 129 ± 9; D2, 144 ± 12; D3, 166 ± 12; D4, 154 ± 10; D5, 168 ± 16). Following each session, the volume of water consumed during the task was supplemented to 1ml if necessary.

For the *goal-directed VR* group, two-photon imaging was performed in combination with a virtual reality system as previously described [71]. Briefly, the task required the mice to lick a spout for a water reward at a specific location along a virtual corridor (120 cm from the beginning of the corridor), which was indicated by a change in visual stimulus from the oriented grating pattern to black walls, referred to as the reward zone. Once the animal entered the reward zone (40 cm total length), within the first 20 cm (120-140cm) it could lick for a water droplet; this was considered a successful trial. When a reward was not triggered by the mouse (unsuccessful trial), animals were given a water droplet at a default location 20 cm beyond the reward zone onset. In phase 1, all animals were presented with a single repetitive grating (vertical oriented bars) on the virtual corridor walls and in phase 2 a subgroup of these animals (5 of 9 mice, see Table S1) were presented with the initial repetitive grating as well as an additional repetitive grating (120 cm total length, 40 cm reward zone) angled at 45° (presented in alternating blocks of 5 trials each). In phase 2, sessions were 30 minutes long with an equal number of trials with each grating.

For the *uncoupled-rewarded* group, animals ran freely and the same grating and black zone were presented on the corridor walls as in the *goal-directed VR* group but visuomotor feedback was uncoupled to optic-flow and varied randomly throughout each session independent of the animals movements (i.e., uncoupled VR playback conditions); therefore, the stimulus temporal frequency was dynamic and randomly varied according to the same range of parameters as in the *goal-directed VR* group (range 0.5-3.5 Hz; average 2 Hz across trials). Mice received rewards by self-initiated licking during the first 1.5 s (successful trial) of the presentation of the reward zone (marked by black corridor walls) or, if this trigger was missed, dispensed by default (unsuccessful trial) after this time.

For the *random reward* group, animals ran freely and the same grating and black zone were presented on the corridor walls as in the *goal-directed VR* group but visuomotor feedback was uncoupled to optic-flow and varied randomly throughout each session independent of the animals movements (i.e., uncoupled VR playback conditions); therefore, the stimulus temporal frequency was dynamic and randomly varied according to the same range of parameters as in the *goal-directed VR* group (range 0.5-3.5 Hz; average 2 Hz across trials). Mice received rewards randomly throughout the session, either during the repetitive stimulus presentation (vertical oriented grating on corridor walls) or during the presentation of the reward zone (marked by black corridor walls).

### Quantification and Statistical Analysis

#### Image analysis

Images resulting from two-photon imaging were analyzed as previously described [27, 71]. Briefly, we used discrete Fourier 2D-based image alignment for motion correction of image frames (SIMA 1.3.2, sequential image analysis [66]). Regions of interest (ROIs) corresponding to neuronal cell bodies were selected manually and aligned across days. Pixel intensity within each ROI was averaged to create a raw fluorescence time series F(t). Baseline fluorescence F_0_ was computed for each neuron by taking the 5^th^ percentile of the smoothed F(t) (1 Hz lowpass, zero-phase, 60^th^-order FIR filter) and the change in fluorescence relative to baseline (ΔF/F_0_) was calculated (F(t)-F_0_/F_0_). In order to remove neuropil contamination, we used nonnegative matrix factorization (NMF), as implemented in FISSA [67] (https://github.com/rochefort-lab/fissa). All further analyses were performed using custom-written scripts in MATLAB (MathWorks, MA, USA), which are freely available via GitHub (https://github.com/rochefort-lab/Henschke_et_al_CurrBiology2020).

#### Licking behavior

To assess the behavioral performance of the mice during the active VR task, a spatial modulation index (SMI) of licking was calculated [71]. The licks of each trial were randomly permuted, and we determined the proportion of trials in which at least one lick event was inside the reward zone. This was repeated 1000 times and the mean success rate of the shuffled distribution was calculated. The SMI value was calculated by dividing the original success rate (successful trials/total number of trials) by the mean of the shuffled distribution. If the animal licks few times but in the right spot, this number will be high (> 1). In contrast, if the animal licks in a spatially indiscriminate pattern, the number will approach 1. If the animal licks often, but keeps missing the reward zone, the SMI will be < 1.

#### Orientation selectivity

The response $r_{t}\left(\theta_{k}\right)$ of a neuron to an oriented grating $\theta_{k}$ during trial t was calculated by averaging the ΔF/F_0_ over the stimulation period. This response was then normalized by subtracting the local baseline activity ($r_{t}\left(\theta_{k}\right)=$ mean ΔF/F_0_ - minimum ΔF/F_0_ during the 2 s window preceding stimulation). The main response $R\left(\theta_{k}\right)$ to orientation $\theta_{k}$ was obtained by averaging the responses $r_{t}\left(\theta_{k}\right)$ across trials. The preferred stimulus of a neuron was the orientation that elicited the maximal response $R\left(\theta_{k}\right)$. The orientation selectivity was characterized by the circular variance (CirVar) [69]:

$$1-CirVar=\left|\frac{\sum_{k}R\left(\theta_{k}\right)\text{exp}\left(2i\theta_{k}\right)}{\sum_{k}R\left(\theta_{k}\right)}\right|$$

The peak angle and peak magnitude of the tuning curve were estimated by taking the argument and modulus of the response vector $\sum_{k}R\left(\theta_{k}\right)\text{exp}\left(2i\theta_{k}\right)/\sum_{k}R\left(\theta_{k}\right)$ [72]. The peak angle was used to compute the orientation shift between pre and post days. A neuron then qualified as orientation selective if it passed two criteria: (i) it was significantly tuned (peak magnitude > 25th percentile of pre testing day for each animal), and (ii) the response to its preferred orientation was significantly higher than to the orthogonal-to-preferred orientation across trials (responses $r_{t}\left(\theta_{k}\right)$, preferred versus orthogonal, across all trials; p < 0.05, Wilcoxon signed rank test).

The reliability of the orientation selectivity was assessed by calculating the coefficient of variation (CV; the ratio of the standard deviation to the mean) of the peak magnitude of the tuning curve across trials.

#### Stimulus decoding and discriminability

To quantify the specific increase in the proportion of neurons selective for the repetitive grating (R_select_ neurons) relative to the change in the proportion of neurons that were selective for the orthogonal grating (O_select_ neurons; which was only presented to the mice on pre and post days but not during the 5 consecutive day) we calculating an index RO_index_ = R_select_-O_select_/R_select_+O_select_ and quantified the change in this ratio from pre to post (post RO_index_ – pre RO_index_). As an additional control, we calculated a similar index (O_index_) between two different oriented gratings (orthogonal to the repetitive, OR_select,_ and orthogonal to the angled grating, OA_select_) both of which, mice were only exposed to on pre and post days (O_index_ = OA_select_-OR_select_/OA_select_+OR_select_).

To quantify the accuracy by which V1 activity could be classified based on the population activity, we used a template-matching decoder [1], which compares the population activity per trial to response templates of the different oriented gratings. These templates are generated by taking the mean ΔF/F_0_ during each oriented stimulus period, or corridor, (θ), for each neuron in a single field-of-view; resulting in a template of population activity (R^θ^) per trial. The similarity of this template to the actual population activity (R^P^) for all other trials per stimulus orientation is given by:

$$I_{\theta }=\frac{\sum_{i=1}^{N}R_{i}^{P}\cdot R_{i}^{\theta }}{\left|R^{\theta }\right|\cdot \left|R^{p}\right|},$$

where *i* indexes the N elements (neurons) of R. The similarity index *I* is calculated for all presented stimulus orientations and the decoded output is determined by taking the condition with the highest similarity to the template population activity. Decoder accuracy is given by the percentage of correctly decoded trials.

To quantify how individual neurons encode information regarding the separate orientated stimuli we used a Bayesian maximum-likelihood decoder for each neuron separately. For each trial, the response of a neuron to a specific orientation was calculated by taking the average ΔF/F_0_ over the 2 s stimulation period. For each orientation θ we approximated the corresponding response distribution of a neuron p(R|θ) with a Gaussian. Leaving one trial out (test trial), we determined the orientation–specific likelihood distribution by computing the mean and standard deviation of the responses to that orientation across the remaining trials (training trials). We then decoded the responses of the test trial; we assumed a uniform prior across orientations and hence the posterior p(θ|R) is directly proportional to the likelihood [1]. Thus, for each response R_t of the test trial we selected the orientation θ that maximized the likelihood p(R_t|θ). We repeated the leave-one-out procedure by looping over trials. The performance for a given neuron was evaluated for each orientation by calculated the percentage of correct inferences. We then averaged the performance for each orientation across all neurons per animal. Finally, we estimated the changes of decoder performance per animal and per orientation between the post and pre testing days.

For Phase 2 of the *goal-directed VR* task, to determine grating responsive neurons (corridor responsive and corridor-selective neurons, i.e neurons that were responsive to the oriented grating stimulus pattern along the virtual corridor), we compared the activity within 25 cm blocks before and after the reward-zone onset for each trial (R_pre_ versus R_post_; p < 0.001, Wilcoxon signed rank test). Corridor responsive neurons were categorized as those where R_pre_ (−35 to −10 cm before the reward-zone onset) was significantly greater than R_post_ (0 to 25 cm after the reward-zone onset), hence, the neuron decreased its activity at the region of the virtual corridor that lacked a visual stimulus (the reward zone), for either, or both, of the presented virtual corridors (vertical and/or angled). Corridor-selective neurons were then further defined as those significantly responsive only to a single virtual corridor (e.g., vertical corridor pattern) and not to the other (e.g., angled corridor pattern).

Stimulus discriminability (d’) was calculated following previously described methods [31, 73] based on the average responses (ΔF/F_0_) across trials for the two behaviorally relevant stimuli (vertical and angled gratings): taking the 2 s stimulation period during the single screen condition and a 2 s period at the start of each trial when each grating was presented on the walls of the virtual corridor for the conditions in the VR environment. The d’ was then calculated as follows:

$$d'=\frac{\mu_{1}-\mu_{2}}{{\left(\frac{1}{2}\left(\sigma_{1}^{2}+\sigma_{2}^{2}\right)\right)}^{\frac{1}{2}}},$$

where μ_1_ and μ_2_ were the means of the responses for each stimulus, and σ^2^ their respective variances and the absolute value taken for analysis.

#### Reward-responsive neurons

To define if a neuron was reward-responsive, we first aligned responses (ΔF/F_0_) to the reward event (i.e., time of reward onset) for each trial. We then examined the average responses within a 1 s window before the onset of the reward event (−2 s from onset to −1 s from onset) compared to the average responses within a 1 s window after the onset of the reward (reward onset to 1 s after reward onset) for each individual trial. If the neuron had significantly higher responses after the reward onset compared to the window before the reward onset it was considered reward-responsive (p < 0.05; paired t test).

#### Analysis of locomotion

For all two-photon systems, locomotion and stationary periods were calculated as previously described [27]. Briefly, stationary periods were defined as time points when the instantaneous speed was < 0.1 cm/s. Locomotion corresponded to periods meeting three criteria: instantaneous speed ≥ 0.1 cm/s, 0.25 Hz lowpass filtered speed ≥ 0.1 cm/s, and an average speed ≥ 0.1 cm/s over a 2 s window centered at the time point. Any inter-locomotion interval shorter than 500 ms was also labeled as locomotion. Periods less than 3 s after or 0.2 s before a period of locomotion were not considered as stationary. We quantified the effect of locomotion on neuronal activity by using a locomotion modulation index (LMI), which is the difference between the ΔF/F_0_ during locomotion (R_L_) and stationary (R_s_) periods, normalized by the activity during both periods: LMI  =  (R_L_-R_s_) ⁄ (R_L_ + R_s_).

#### Statistics

Unless otherwise stated, error bars in all graphs indicate standard error of the mean (s.e.m.) and all statistical tests were two-tailed. Unless otherwise stated, mean, error values and statistics were calculated across animals; for analysis where we isolated specific populations of neurons with multiple response parameters across experimental groups (e.g., for Figure 6), mean and error values and statistics were calculated across neurons. For planned comparisons across different experimental groups, we used one-way ANOVA with Fisher’s least significant difference (lsd) test with no correction for multiple comparisons. Since, in our study design, we have formulated a specific hypothesis for the tests we perform across specific experimental groups, we are testing these planned comparison predictions using the lsd test to increase our statistical power and avoid the increased probability of Type II errors that may occur with other post hoc tests [74]; this choice is at the cost of potentially increasing the likelihood of Type 1 errors. We report exact p values for all the comparisons that were made in Table S2. For paired comparisons across conditions or days for the same population of neurons, or when assessing the proportion of neurons within each group, we used the Wilcoxon signed rank test (non-parametric paired difference test) or paired t test. For unpaired comparisons across days involving different underlying population of neurons within the same group, we used Mann-Whitney U-test or unpaired Student’s t test. For all correlations we report Pearson’s correlation coefficient.

### Data and Code Availability

The code used for analysis in this study are freely available via GitHub repository (https://github.com/rochefort-lab/Henschke_et_al_CurrBiology2020).

## Supplemental Information

Document S1. Figures S1–S3 and Tables S1 and S2

Document S2. Article plus Supplemental Information
