# Supplementary material for: Reward Association Enhances Stimulus-Specific Representations in Primary Visual Cortex
Source: Curr Biol. 2020 May 18;30(10):1866–1880.e5. doi: 10.1016/j.cub.2020.03.018 (PMC7237886; doi:10.1016/j.cub.2020.03.018)
Supplement: Document S1. Figures S1–S3 and Tables S1 and S2 [file mmc1.pdf]

Current Biology, Volume *30*

Supplemental Information

Reward Association Enhances Stimulus-Specific Representations in Primary Visual Cortex

Julia U. Henschke, Evelyn Dylda, Danai Katsanevaki, Nathalie Dupuy, Stephen P. Currie, Theoklitos Amvrosiadis, Janelle M.P. Pakan, and Nathalie L. Rochefort

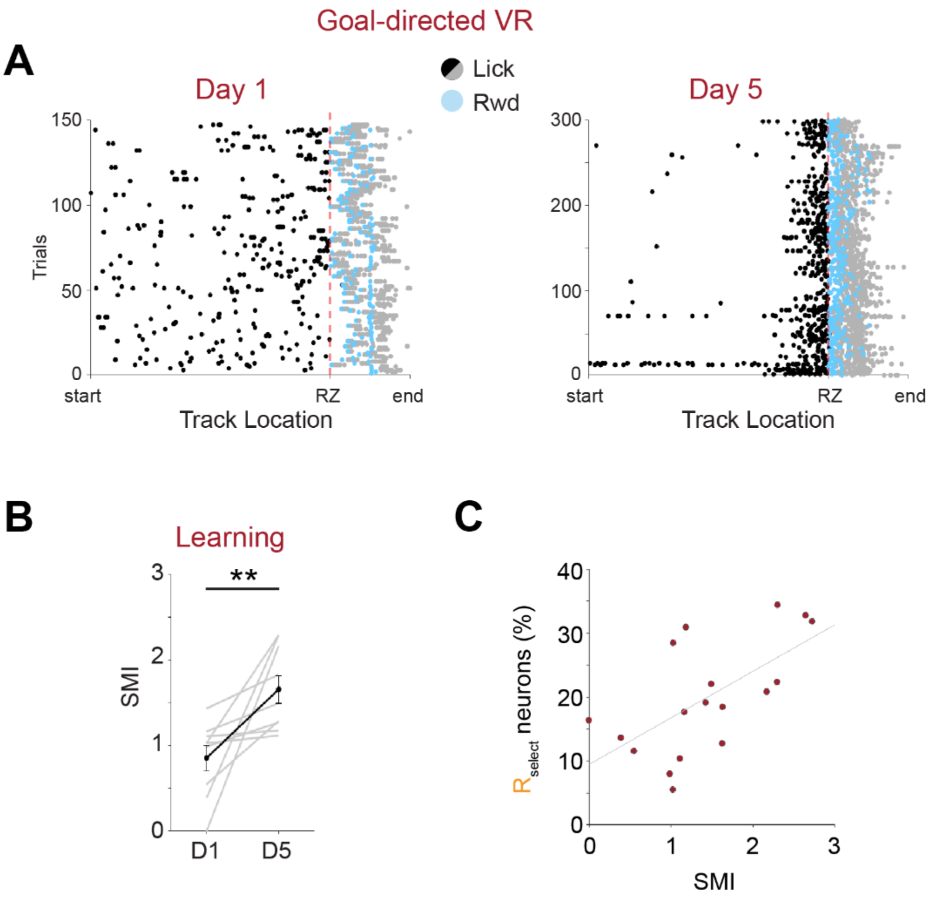

Figure S1. Licking behaviour during the repetitive stimulus presentation in a virtual environment, Related to Figure 1.

(A) Raster plots of licking behavior during the goal-directed VR task for a single mouse on day 1 (D1; left) and day 5 (D5; right) of the repetitive stimulus presentation. Licks along the virtual corridor that occur before the reward is given (black), the lick that triggers the water reward (blue) and the licks following the reward (i.e. drinking behaviour; grey) are shown. Rewards triggered in the first half of the reward zone (RZ; start indicated by red dashed line) are successful trials.

(B) Task performance for each animal (grey) in the goal-directed VR group is shown as the spatial modulation index (SMI; success rate/proportion of trials that would be successful with a shuffled distribution of licking), with mean SMI across all animals shown in black. A larger SMI indicates that the animal was licking in a spatially discrete area surrounding the reward zone (SMI: D1 to D5, $p = 0.004$; $n = 9$ mice; Wilcoxon signed rank test).

(C) Correlation between the proportion of neurons orientation selective for the repetitive grating (R_{select}) on either Pre testing day or Post testing day and behavioral performance quantified by SMI on novice training day (day 1) or at peak performance (max SMI day 2-5), respectively ($R = 0.627$, $p = 0.005$, $n = 18$ [Pre and Post day from 9 mice]; Pearson's coefficient).

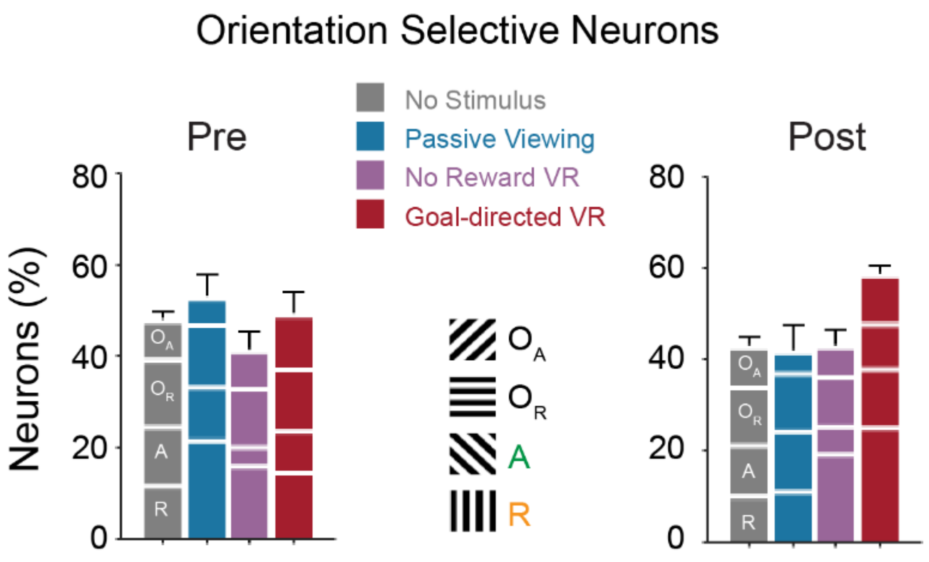

Figure S2. Proportion of orientation selective neurons. Related to Figure 1.
Percentage of orientation selective neurons is shown for each experimental group. Stacked divisions indicate the proportion of selective neurons for each oriented stimulus. The repetitive stimulus (R; vertical oriented bars) is on the bottom, followed by the angled stimulus (A; R-45°), then the orthogonal to repetitive stimulus (OR; horizontally oriented bars; R+90°), and finally, the orthogonal to angled stimulus (AR; R+45°) on top.

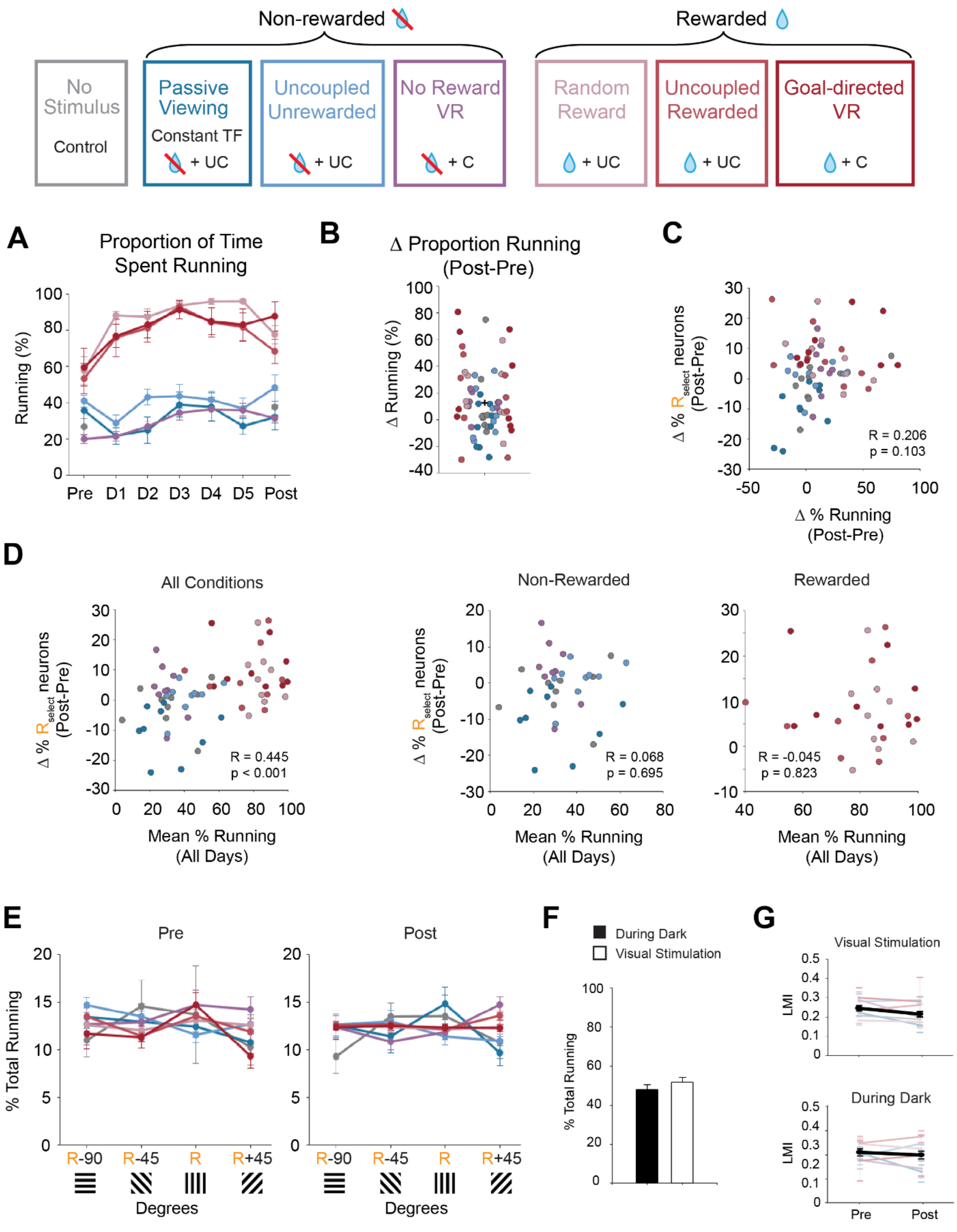

Figure S3. Locomotion does not correlate to differences in visual responses within each experimental group, Related to Figure 2.

Experimental groups depending on the presence or absence of reward and coupled (C) or uncoupled (UC) visuomotor feedback during the repetitive visual stimulus over 5 days are shown; rewarded and non-rewarded groups indicated. Group colors apply to all panels.

(A) Proportion of time spent running for day 1 (D1) through to day 5 (D5) of the repetitively presented stimulus and before (Pre) compared to after (Post) testing days for each group.

(B) Change in the proportion of time spent running between Post and Pre testing days for all experimental groups.

(C) Correlation between the change in the proportion of R_{select} neurons from Pre to Post day and the change in the proportion of time spent running from Pre to Post day ($R = 0.206$, $p = 0.103$, Pearson's coefficient).

(D) Correlation between the change in the proportion of R_{select} neurons from Pre to Post day and the average proportion of time spent running across all experimental days for each experimental group. Although there was a significant correlation across all conditions ($R = 0.445$, $p < 0.001$, $n = 63$; Pearson's coefficient), there were no significant correlations within any group (no stimulus: $R = 0.086$, $p = 0.826$; passive-viewing: $R = -0.050$, $p = 0.899$; uncoupled-unrewarded: $R = 0.455$, $p = 0.218$; no reward VR: $R = -0.477$, $p = 0.194$; random reward: $R = -0.118$, $p = 0.762$; uncoupled-unrewarded: $R = 0.106$, $p = 0.786$; goal-directed VR: $R = -0.213$, $p = 0.583$; Pearson's coefficient). There was also no significant correlation for non-rewarded (including control; $R = 0.068$, $p = 0.695$, $n = 36$; Pearson's coefficient) or rewarded ($R = -0.045$, $p = 0.823$, $n = 27$; Pearson's coefficient) conditions separately.

(E) Proportion of time spent running during the presentation of each oriented stimulus for the Pre and Post testing days. Repetitive grating (R). There was no statistically significant interaction between the orientation and testing day on the proportion of locomotion ($p = 0.828$, two-way ANOVA).

(F) Proportion of time spent running in the dark, versus during visual stimulation was not significantly different across all conditions ($p = 0.230$, one-way ANOVA).

(G) Change in locomotion modulation index (LMI) from Pre to Post day for all experimental groups during both visual stimulation and during darkness. No significant difference across Pre and Post testing days (during visual stimulation, $p = 0.169$, during dark, $p = 0.887$, one-way ANOVA)

Groups and animals	Repetitive stimulation			Gender	System	Frame Rate (Hz)	Objective	Pixel size (µm)	FOV (mm^2)	ROIs	Average density (ROIs/mm^2)
	TF	Visuomotor feedback	Reward								
**No stimulus**											
**1**	N/A	N/A	None	M	Custom	40	40	0.40	0.05	72	
**2**	N/A	N/A	None	F	Custom	40	25	0.64	0.12	160	
**3**	N/A	N/A	None	F	Custom	40	25	0.64	0.12	163	
**4**	N/A	N/A	None	M	Custom	40	25	0.64	0.12	262	
**5**	N/A	N/A	None	M	Custom	40	40	0.40	0.05	126	
**6**	N/A	N/A	None	M	Custom	40	25	0.64	0.12	193	
**7**	N/A	N/A	None	M	Custom	40	25	0.64	0.12	215	
**8**	N/A	N/A	None	M	Custom	40	25	0.64	0.12	118	
**9**	N/A	N/A	None	M	Custom	40	25	0.64	0.12	135	
Total									0.94	1444	1536
**Passive Viewing**											
**1**	Constant	Uncoupled	None	M	Custom	40	40	0.40	0.04	92	
**2**	Constant	Uncoupled	None	F	Custom	40	40	0.40	0.05	91	
**3**	Constant	Uncoupled	None	F	Custom	40	40	0.40	0.04	102	
**4**	Constant	Uncoupled	None	F	Custom	40	25	0.64	0.12	180	
**5**	Constant	Uncoupled	None	M	Custom	40	25	0.64	0.12	209	
**6**	Constant	Uncoupled	None	M	Custom	40	40	0.40	0.04	97	
**7**	Constant	Uncoupled	None	M	Custom	40	25	0.64	0.12	294	
**8**	Constant	Uncoupled	None	M	Custom	40	40	0.40	0.05	87	
**9**	Constant	Uncoupled	None	M	Custom	40	40	0.40	0.05	93	
Total									0.63	1245	1976
**Uncoupled unrewarded**											
**1**	Dynamic	Uncoupled	None	F	Custom	40	25	0.64	0.12	84	
**2**	Dynamic	Uncoupled	None	F	Custom	40	25	0.64	0.12	108	
**3**	Dynamic	Uncoupled	None	F	Custom	40	25	0.64	0.12	141	
**4**	Dynamic	Uncoupled	None	M	Custom	40	25	0.64	0.12	132	
**5**	Dynamic	Uncoupled	None	M	Custom	40	25	0.64	0.12	188	
**6**	Dynamic	Uncoupled	None	M	Custom	40	25	0.64	0.12	157	
**7**	Dynamic	Uncoupled	None	M	Custom	40	25	0.64	0.12	154	
**8**	Dynamic	Uncoupled	None	F	Custom	40	25	0.64	0.12	110	
**9**	Dynamic	Uncoupled	None	F	Custom	40	25	0.64	0.12	113	
Total									1.08	1187	1099
**Passive VR**											
**1**	Dynamic	Coupled	None	F	B-Scope	30	20	0.85	0.19	57	
**2**	Dynamic	Coupled	None	F	B-Scope	30	20	0.85	0.19	440	
**3**	Dynamic	Coupled	None	F	B-Scope	30	20	0.85	0.19	500	
**4**	Dynamic	Coupled	None	F	B-Scope	30	20	0.85	0.19	281	
**5**	Dynamic	Coupled	None	F	B-Scope	30	20	0.85	0.19	585	
**6**	Dynamic	Coupled	None	F	B-Scope	30	20	0.85	0.19	237	
**7**	Dynamic	Coupled	None	F	B-Scope	30	20	0.85	0.19	423	
**8**	Dynamic	Coupled	None	F	B-Scope	30	20	0.85	0.19	400	
**9**	Dynamic	Coupled	None	F	B-Scope	30	20	0.85	0.19	416	
Total									1.71	3339	1952
**Random reward**											
**1**	Dynamic	Uncoupled	Random	M	HyperScope	30	16	0.75	0.15	99	

**2**	Dynamic	Uncoupled	Random	M	HyperScope	30	16	0.75	0.15	320	
**3**	Dynamic	Uncoupled	Random	M	HyperScope	30	16	0.75	0.15	252	
**4**	Dynamic	Uncoupled	Random	M	HyperScope	30	16	0.75	0.15	242	
**5**	Dynamic	Uncoupled	Random	M	HyperScope	30	16	0.75	0.15	315	
**6**	Dynamic	Uncoupled	Random	F	HyperScope	30	16	0.75	0.15	206	
**7**	Dynamic	Uncoupled	Random	F	HyperScope	30	16	0.75	0.15	69	
**8**	Dynamic	Uncoupled	Random	F	HyperScope	30	16	0.75	0.15	189	
**9**	Dynamic	Uncoupled	Random	M	HyperScope	30	16	0.75	0.15	121	
Total									1.35	1813	1342
**Uncoupled rewarded**											
**1**	Dynamic	Uncoupled	RZ	M	HyperScope	30	16	0.75	0.15	120	
**2**	Dynamic	Uncoupled	RZ	M	HyperScope	30	16	0.75	0.15	338	
**3**	Dynamic	Uncoupled	RZ	M	HyperScope	30	16	0.75	0.15	284	
**4**	Dynamic	Uncoupled	RZ	M	HyperScope	30	16	0.75	0.15	254	
**5**	Dynamic	Uncoupled	RZ	M	HyperScope	30	16	0.75	0.15	347	
**6**	Dynamic	Uncoupled	RZ	F	HyperScope	30	16	0.75	0.15	211	
**7**	Dynamic	Uncoupled	RZ	F	HyperScope	30	16	0.75	0.15	76	
**8**	Dynamic	Uncoupled	RZ	M	Custom	40	25	0.64	0.12	203	
**9**	Dynamic	Uncoupled	RZ	F	Custom	40	25	0.64	0.12	175	
Total									1.29	2008	1556
**Goal-directed VR**											
**1**	Dynamic	Coupled	RZ	F	Custom	40	25	0.64	0.12	218	
**2**	Dynamic	Coupled	RZ	F	Custom	40	25	0.64	0.12	159	
**3**	Dynamic	Coupled	RZ	F	Custom	40	25	0.64	0.12	58	
**4**	Dynamic	Coupled	RZ	F	Custom	40	25	0.64	0.12	110	
**5***	Dynamic	Coupled	RZ	M	Custom	40	40	0.40	0.05	87	
**6***	Dynamic	Coupled	RZ	M	Custom	40	40	0.40	0.05	55	
**7***	Dynamic	Coupled	RZ	M	Custom	40	40	0.40	0.05	103	
**8***	Dynamic	Coupled	RZ	M	Custom	40	40	0.40	0.05	63	
**9***	Dynamic	Coupled	RZ	M	Custom	40	25	0.64	0.12	202	
Total									0.8	1055	1318
Grand Total									7.8	12091	Avg: 1540

Table S1. Stimulus presentation and imaging parameters for each experimental group and each animal within each group, Related to STAR Methods. TF; temporal frequency of repetitive stimulus presentation. M: male; F: female. RZ: reward given in reward zone demarcated by black corridor walls in virtual environment. FOV: field-of-view. ROIs: regions of interest, indicating how many neurons were analysed within a FOV for each mouse. *Mice also used for phase 2 of *goal-directed VR* task (see Figure 4 and Figure 5).

Figure	Measure	Values	Omnibus	Comparisons
1E	Δ RO_{index}	*no stimulus*: 0.023 ± 0.099; *passive viewing*: -0.232 ± 0.101; *no reward VR*: 0.083 ± 0.153; *goal-directed VR:* 0.529 ± 0.118	one-way ANOVA, p = 0.001	*no stimulus vs passive viewing:* p = 0.147; *no stimulus vs no reward VR*: p = 0.728; *no stimulus vs goal-directed VR*: p = 0.006; *passive viewing vs no reward VR*: p = 0.075; *passive viewing vs goal-directed VR*: p = 0.0001; *no reward VR vs goal-directed VR*: p = 0.014
1F	Orientation Shift (degrees)	*no stimulus*: 0.423 ± 2.591; *passive viewing*: -5.636 ± 1.903; *no reward VR*: 0.320 ± 3.377; *goal-directed VR:* 13.806 ± 2.926	one-way ANOVA, p = 0.0002	*no stimulus vs passive viewing*: p = 0.129; *no stimulus vs no reward VR*: p = 0.979; *no stimulus vs goal-directed VR*: p = 0.002; *passive viewing vs no reward VR*: p = 0.136; *passive viewing vs goal-directed VR*: p = 0.00002; *no reward VR vs goal-directed VR*: p = 0.002
1G	Δ Decoder Accuracy (%)	*no stimulus*: -2.2 ± 1.7 %; *passive viewing*: -4.1 ± 1.6 %; *no reward VR*: 2.4 ± 1.3 %; *goal-directed VR*: 10.7 ± 2.9 %	one-way ANOVA, p = 0.0001	*no stimulus vs passive viewing*: p = 0.417; *no stimulus vs no reward VR*: p = 0.048; *no stimulus vs goal-directed VR*: p = 0.000115; *passive viewing vs no reward VR*: p = 0.00074; *passive viewing vs goal-directed VR*: p = 0.00002; *no reward VR vs goal-directed VR*: p = 0.00076
2B	Δ Selective Neurons for repetitive grating (%)	*no stimulus*: -2.1 ± 2.4 %; *random reward*: 7.0 ± 3.1 %; *uncoupled rewarded*: 7.5 ± 3.3 %; *goal-directed VR*: 10.6 ± 2.7 %	one-way ANOVA, p = 0.023	*no stimulus vs random reward:* p = 0.032; *no stimulus vs uncoupled rewarded*: p = 0.025; *no stimulus vs goal-directed VR*: p = 0.004; *random reward vs uncoupled rewarded*: p = 0.911; *random reward vs goal-directed VR*: p = 0.378; *uncoupled rewarded vs goal-directed VR*: p = 0.440
2C	Δ RO_{index}	*no stimulus*: 0.023 ± 0.099; *random reward*: 0.341 ± 0.232; *uncoupled rewarded*: 0.504 ± 0.212; *goal-directed VR:* 0.529 ± 0.118	one-way ANOVA, p = 0.032	*no stimulus vs random reward:* p = 0.174; *no stimulus vs uncoupled rewarded*: p = 0.019; *no stimulus vs goal-directed VR*: p = 0.028; *random reward vs uncoupled rewarded*: p = 0.911; *random reward vs goal-directed VR*: p = 0.416; *uncoupled rewarded vs goal-directed VR*: p = 0.748
2D	Orientation Shift (degrees)	*no stimulus*: 0.423 ± 2.591; *random reward*: 7.316 ± 3.066; *uncoupled rewarded*: 9.556 ± 3.930; *goal-directed VR:* 13.806 ± 2.926	one-way ANOVA, p = 0.039	*no stimulus vs random reward:* p = 0.134; *no stimulus vs uncoupled rewarded*: p = 0.049; *no stimulus vs goal-directed VR*: p = 0.005; *random reward vs uncoupled rewarded*: p = 0.620; *random reward vs goal-directed VR*: p = 0.157; *uncoupled rewarded vs goal-directed VR*: p = 0.350
2E	Δ Decoder Accuracy (%)	*no stimulus*: -2.2 ± 1.7 %; *random reward*: 4.7 ± 1.8 %; *uncoupled rewarded*: 3.9 ± 1.7 %; *goal-directed VR*: 10.7 ± 2.9 %	one-way ANOVA, p = 0.003	*no stimulus vs random reward:* p = 0.010; *no stimulus vs uncoupled rewarded*: p = 0.021; *no stimulus vs goal-directed VR*: p = 0.0004; *random reward vs uncoupled rewarded*: p = 0.749; *random reward vs goal-directed VR*: p = 0.072; *uncoupled rewarded vs goal-directed VR*: p = 0.043
3D	Factor Δ in Selectivity Magnitude	*no stimulus*: 0.932 ± 0.070; *passive viewing*: 0.938 ± 0.028; *unrewarded uncoupled*: 0.931 ± 0.031; *no reward VR*: 0.958 ± 0.028; *random reward*: 1.060 ± 0.041; *uncoupled rewarded*: 1.048 ± 0.069; *goal-directed VR:* 1.187 ± 0.070	one-way ANOVA, p = 0.006	*no stimulus vs passive viewing:* p = 0.930; *no stimulus vs uncoupled unrewarded:* p = 0.994; *no stimulus vs no reward VR*: p = 0.695; *no stimulus vs random reward*: p = 0.064; *no stimulus vs uncoupled rewarded*: p = 0.105; *no stimulus vs goal-directed VR*: p = 0.001; *goal-directed VR vs passive viewing*: p = 0.0008; *goal-directed VR vs uncoupled unrewarded*: p = 0.0009; *goal-directed VR vs no reward VR*: p = 0.002; *goal-directed VR vs random reward*: p = 0.082; *goal-directed VR vs uncoupled rewarded*: p = 0.065
3D	Factor Δ in Variability Across Trials	*no stimulus*: 1.037 ± 0.032; *passive viewing*: 0.981 ± 0.031; *unrewarded uncoupled*: 0.985 ± 0.018; *no reward VR*: 0.969 ± 0.021; *random reward*: 0.924 ± 0.011; *uncoupled rewarded*: 0.919 ± 0.015; *goal-directed VR:* 0.898 ± 0.017	one-way ANOVA, p = 0.0004	*no stimulus vs passive viewing:* p = 0.082; *no stimulus vs uncoupled unrewarded:* p = 0.094; *no stimulus vs no reward VR*: p = 0.031; *no stimulus vs random reward*: p = 0.0005; *no stimulus vs uncoupled rewarded*: p = 0.0003; *no stimulus vs goal-directed VR*: p = 0.000003; *goal-directed VR vs passive viewing*: p = 0.011; *goal-directed VR vs uncoupled unrewarded*: p = 0.006; *goal-directed VR vs no reward VR*: p = 0.024; *goal-directed VR vs random reward*: p = 0.409; *goal-directed VR vs uncoupled rewarded*: p = 0.508

Table S2. Summary of data and exact p-values, Related to Figure 1, 2, and 3. Applicable Figure panel is listed followed by the relevant measure for which data is reported. Data values are given as mean ± standard error (s.e.m.). Exact p-values for omnibus statistics (one-way ANOVAs) are then followed by p-values for comparisons made for each Figure panel (Fisher's least significant difference (lsd) test with no correction for multiple comparisons). For panels from Figure 1 and 2, planned comparisons were made across selected 3-4 of seven experimental groups included in the respective Figure panels. For comparisons in Figure 3D including all seven experimental groups, planned comparisons were made between control (*no stimulus* group) versus other groups, as well as the active task-learning group (*goal-directed VR*) versus other groups. See also STAR methods.
